# Neural correlates of different self domains

**DOI:** 10.1002/brb3.409

**Published:** 2015-10-21

**Authors:** Helder F. Araujo, Jonas Kaplan, Hanna Damasio, Antonio Damasio

**Affiliations:** ^1^ Brain and Creativity InstituteUniversity of Southern CaliforniaLos AngelesCalifornia; ^2^ Neuroscience Graduate ProgramUniversity of Southern CaliforniaLos AngelesCalifornia; ^3^ Graduate Program in Areas of Basic and Applied BiologyUniversity of OportoOportoPortugal

**Keywords:** Autobiographical self, core self, exteroception, interoception, memory, self

## Abstract

**Introduction:**

The neural substrates of states devoted to processing self‐related information (“self‐related states”) remain not fully elucidated. Besides the complexity of the problem, there is evidence suggesting that self‐related states vary according to the information domain being considered. Here, we investigated brain correlates for self‐related states concerning historical aspects of one's life (autobiographical self), and one's ongoing body status (core self). We focused on memory‐related regions, body‐related regions, CMSs (cortical midline structures), and ICs (insular cortices).

**Methods:**

This was a block‐design fMRI study contrasting brain activity for core self (interoception and exteroception) and autobiographical self (personality traits and biographic facts) information domains. It involved 19 participants, who answered questions about each domain (four conditions).

**Results:**

All conditions appeared to engage the regions of interest. Nonetheless, autobiographical self compared with core self showed greater activity in memory‐related regions (e.g., hippocampus), MPFC (medial prefrontal cortex), superior PMC (posteromedial cortex), and anterior ICs. Core self compared with autobiographical self was associated with greater activity in body‐related regions (e.g., somatosensory cortices, and EBA [extrastriate body area]), superior PMC, and posterior ICs. In addition, (1) facts compared with traits showed greater activity in body‐related regions, memory‐related regions, MPFC, and PMC; (2) traits compared with facts were associated with greater activity in the posterior part of the anterior cingulate cortex; (3) interoception compared with exteroception was associated with greater activity in body‐related regions (e.g. postcentral gyrus), memory‐related regions, MPFC, inferior PMC and ICs; (4) exteroception compared with interoception showed greater activity in some body‐related regions (e.g., premotor cortices and EBA) and superior PMC.

**Conclusions:**

The results support the notion that the neural correlates of self‐related states depend on the information domain. Those states seem distinguishable in terms of activity in memory‐related and body‐related regions, and activity in regions that have been associated with self processes (CMSs and the ICs).

## Introduction

The neural bases for the self remain to be fully elucidated. A great deal of the relevant investigation has focused on the comparison between self‐related states (e.g., processing information related to oneself, “self”) and nonself‐related states (e.g., processing information related to another person, “other”). This research has been informative (for a review, e.g., Northoff et al. 2006). For example, it is known that processing personality traits tends to yield different levels of brain activity for self than for other. Specifically, the MPFC (medial prefrontal cortex) tends to be more active for self than other, and the PMC (posteromedial cortex) tends to be more active for other than for self (Araujo et al. 2013). However, determining differences between self and other addresses only part of the problem. There is increasing evidence that self states are not unitary. Instead, they vary depending on the specific self information domain that is being considered (Klein 2012). In a previous study, for instance, we demonstrated that evaluating one's personality traits is associated with different brain activity from the one found during the evaluation of autobiographic facts (Araujo et al. 2014). In current study, we expanded on those findings and contrasted brain activity for different domains of self information.

We focused on two kinds of self‐related mental states: states pertaining to historical aspects of one's life, and states pertaining to one's ongoing body status. The first kind of mental states have been designated as *autobiographical self* (Damasio, 1998), or narrative self (Gallagher, 1999). They are generated when individuals retrieve memories for historical aspects of their lives, and thus are dominated by biographical information, including simple facts of one's identity (e.g., date and place of birth), personality traits (e.g., honesty), as well as specific life events and episodes (e.g., one's high school graduation). The second kind of mental states may be designated as *core self* (Damasio, 1998). Such states allow individuals to form an account of their ongoing body states, and may relate to interoceptive body changes (e.g., hunger, thirst, or fatigue), and to a class of exteroceptive changes caused by the interaction of the body with the outside world (e.g., pressure exerted on one's arm).

Given the conceptual differences between those kinds of self states, we were interested in investigating activity in brain regions related to memory processes (e.g., hippocampus) and related to body processes (e.g., somatosensory cortices). We focused also on two sets of brain regions that have been associated with self processes: the cortical midline structures (e.g., Northoff et al. 2006), and the ICs (insular cortices) (e.g., Craig 2002).

There is evidence that CMSs (cortical midline structures), particularly the MPFC and the PMC, are engaged when individuals examine aspects of their personalities or their identities (Northoff and Bermpohl 2004), suggesting that CMSs play a role in autobiographical self states. Still, existing studies indicate that CMSs are not dedicated to autobiographical self and assist a wider range of internally oriented processes (Araujo et al. 2013) including those behind core self states. Moreover, the CMSs are highly connected to cortical and subcortical regions related to processing body information (Parvizi et al. 2006; Hagmann et al. 2008). It is thus important to investigate how activity in CMSs differs for autobiographical self and core self.

The ICs have been shown to be involved in processing varied body sensations, especially those related to interoception (Craig 2002), raising the possibility that the insula also plays a role in generating core self states. But there is also evidence, albeit more limited, that the ICs are involved in memory retrieval (Singer et al. 2009) and in evaluating one's personality traits (Modinos et al. 2009), indicating that the ICs would also contribute to autobiographical self mental states. As in the case of CMSs, it is important to determine how the involvement of the insula differs for autobiographical self and core self.

This study is an fMRI study in which participants were asked to answer questions about themselves. The questions required that the participants examined aspects related to their personality and biography (mental states pertaining to the autobiographical self), or aspects related to their ongoing body status (mental states pertaining to the core self). In the hope of ensuring that participants would disengage from self‐related examination during baseline, we used an active baseline consisting of periods of one‐back task in a block design.

The autobiographical self questions were also organized into two experimental conditions, one concerning personality traits (“traits”; e.g., “Does the word honest describe you?”); another, critical biography and identity facts (“facts”; e.g., “Are you a student?”). In both conditions, individuals needed to examine historical aspects of themselves, but each condition focused on a separate domain of autobiographical self given that personality traits and biographical facts are distinct (Keenan et al. 1992; Araujo et al. 2014). Personality traits vary in valence and desirability, whereas biographic facts tend to vary less in that regard; moreover, the examination of one's personality traits is relatively subjective because it depends on personal judgments of a set of experiences, while the examination of one's biographical facts is largely objective because such facts tend to be incontrovertible and verifiable (Keenan et al. 1992; Araujo et al. 2014).

The core self questions were organized into two experimental conditions, according to the domains of body sensations targeted by the questions: (1) the internal milieu, “interoception” (e.g., “Do you feel hungry?”); or (2) skin contact with external stimuli, “exteroception” (e.g., “(e.g., “Do your legs feel wet?”).”). Both conditions required that individuals examine their ongoing body status, but distinct core self domains were targeted because there are substantial physiological differences between interoception and exteroception (Kandel et al. 2013).

We hypothesized that autobiographical self and core self condition should vary according to activity generated in the four sets of ROI (regions of interest) mentioned above: (1) memory‐related brain regions; (2) body‐related brain regions; (3) CMSs; and (4) ICs. Specifically, we predicted the following:

1. Memory‐related brain regions should show greater level of activity for autobiographical self conditions than for core self conditions because autobiographical self states required greater level of memory‐related processing than core self states. We note that the memories retrieved to answer the autobiographical self may be relatively simple and hold qualities of semantic memory (e.g., summary representations) or be more complex and episodic (“exemplars”). Still, core self conditions are likely to elicit some memory retrieval. For example, it has been shown that interoceptive sensations can be effective memory cues (Hirsh 1974). Accordingly, core self conditions may be associated with activity in memory‐related regions but to a lesser extent and degree compared with autobiographical self conditions.
2. Body‐related brain regions should reveal a greater level of activity for core self conditions than for autobiographical self conditions because core self states require greater level of body‐related processing than autobiographical self states. Nonetheless, autobiographical self conditions may be associated with some processing of body‐related representations; such processing should relate predominantly to emotional responses elicited by memory retrieval and decision processes required to answer autobiographical self questions, and is thus associated with activity in regions supporting emotion‐related somatic representations, such as the insular and anterior cingulate cortices.
3. CMSs should be involved in both core and autobiographical self states, but their involvement should be greater for autobiographical self conditions than for core self conditions. As postulated before, we believe that the level generated in CMSs is commensurable with the level of processing of internally generated representations (Araujo et al. 2013). The level of such processing is likely to be greater for memories than for body sensations. Memory retrieval is an elaborative process and requires assembling of a variable number of representations for the different elements of a given memory. We note that, even though the retrieval of certain memories (e.g., semantic memories) may be relatively simple, retrieving a memory tends to co‐evoke related memories. Moreover, even relatively simple memories are likely to be associated with varied imagery pertaining to different sensory modalities. On the other hand, although certain body sensations are associated with relatively varied mental imagery, such as auditory imagery (e.g., shortness of breath) and visual imagery (e.g., pallor associated with nausea), the scope of the imagery for body sensations is likely to be more limited (Critchley and Harrison 2013). Certain regions within CMSs, such as the cingulate cortex and the superior part of the precuneus, should be more active for core self than for autobiographical self because they are strongly connected with regions involved in body processes (Parvizi et al. 2006; Cameron 2009). Moreover, the cingulate cortex is connected to brainstem nuclei related to interoception (Cameron 2009) and is thus possibly more active for interoception than for exteroception. Likewise, the superior precuneus is predominantly linked to somatosensory, motor and premotor cortices (Parvizi et al. 2006), and is likely to be more active during exteroception than during interoception.
4. The ICs should be involved in core self and autobiographical self states, but we predict that the anterior IC is more active for autobiographical self states than for core self states; the reverse pattern applies to the posterior IC. We base this prediction on findings suggesting that body‐related processing is relatively simple in the posterior IC and relates to “actual” body changes, but it becomes increasingly more complex and related to processing of affective responses in the anterior ICs (see Craig 2002, 2009).

## Methods

### Participants

Twenty participants (10 female, and 10 male; 22.5 ± 2.6 years old) were recruited from the University of Southern California community. All participants were native English speakers, right‐handed, with no history of neurological diseases. They were paid for their participation, and provided written informed consent following the Institutional and Federal Guidelines. The data from one of the participants were excluded because he was not able to read some of the questions due to uncorrected myopia. The final study sample consisted of 19 participants (10 female, and nine male; 22.6 ± 2.6 years old).

### Materials and procedures

The experimental stimuli consisted of questions that varied according to four experimental conditions: (1) *traits*, regarding one's personality traits (e.g., “Does the word ‘honest’ describe you?”); (2) *facts*, regarding objective biographical facts, such as demographic data (e.g., “Are you a student?”); (3) *interoception*, regarding sensations pertaining to internal aspects of one's body (e.g., “Do you feel hungry?”); and (4) *exteroception*, regarding sensations pertaining to external aspects of one's body (e.g., “Do your legs feel wet?”).

The questions about personality traits contained a selection of personality traits from a list of personality traits rated in terms of likableness and meaningfulness by 100 college students (Anderson, 1968). The selection included only adjectives with the highest meaningfulness scores and equal numbers of negative traits (the least liked adjectives) and positive traits (the most liked adjectives) The questions about biographic facts covered several aspects of one's life, such as age, height, weight, ethnicity, nationality, occupation, typical means of transportation, household and physical appearance. In both sets (traits and biographic facts), the questions were the same as those used in Araujo et al. 2014.

The questions about interoception involved interoceptive sensations related to one's mouth, nose and throat (e.g., ‘Is your throat OK?’), gastrointestinal system (“Does your stomach ache?’), and heartbeat and respiratory movements, as well as questions related to absence or presence hunger or thirst (e.g., “Do you feel thirsty?”). The questions about exteroception comprised sensations regarding pressure, dryness or wetness, in relation to the neck and back, upper and lower limbs (e.g., “Can you feel anything touching your legs?”; “Do your arms feel dry?”); The participants were instructed that questions in those two conditions (interoception and exteroception) referred to current body sensations (i.e., sensations occurring at the moment they read the question).

The participants answered the questions with “yes” or “no”. A third option (“other”) was available when the participants did not know the correct answer, or did not consider “yes” or “no” as the correct answer.

The baseline separating the blocks of questions consisted of periods of one‐back task, during which the participants saw a series of letters, one at a time, and had to decide whether each of the presented letters was identical to the one immediately preceding it.

All stimuli were presented visually on a screen at the end of the scanner bore, viewed through a mirror placed on the head coil. The participants responded to the stimuli by pressing a button with their right‐hand fingers. We used MATLAB (The Mathworks) RRID:nlx_153890 and Psychophysics Toolbox Version 3 (The Psychophysics Toolbox, 2001) RRID:rid_000041 for both the stimulus presentation and the response collection.

The study comprised three functional runs. Each run lasted 9.7 min and was organized in a block design, containing three blocks for each condition. A block lasted 24 sec and included six questions. Each question was presented for 4 sec. Blocks of questions were separated from one another by a 24‐sec block of the one‐back‐task. The blocks were presented in a randomized order for each run and each participant.

After the scanning, the participants estimated the number of memory episodes they recalled in order to answer the questions in each autobiographical self condition, using a 7‐point Likert scale (1: *I did not recall any particular memory episode.;* 7: *I recalled many episodes…*). In addition, they estimated the number of memory episodes elicited by the questions in each core self conditions, using a Likert scale that ranged from 1 (*I did not recall any memory episodes*) to 7 (*I recalled many memory episodes*).

### Image acquisition

The magnetic resonance images were acquired with a 3‐Tesla Siemens MAGNETON Trio System (Siemens Medical Solutions, Erlangen, Germany). The acquisition of echo‐planar images was performed using the following parameters: TR = 2000 msec, TE = 25 msec, flip angle = 90°, 64 × 64 matrix, in‐plane resolution 3.0 mm × 3.0 mm, 41 transverse slices, each 3 mm thick, and field of view covering the whole brain. Each run consisted of 291 volumes. The acquisition of the structural images (T1‐weighted magnetization‐prepared rapid gradient echo, MPRAGE) used the following parameters: TR = 1950 msec, TE = 2.3 msec, flip angle = 7°, 256 × 256 matrix, 193 coronal slices, 1 mm isotropic resolution.

### Image processing and analysis

The functional imaging data were preprocessed, registered, and analyzed with FSL RRID:birnlex_2067 (FMRIB's Software Library, www.fmrib.ox.ac.uk/fsl). The preprocessing included the following steps: (1) motion correction with MCFLIRT (Jenkinson et al. 2002); (2) slice‐timing correction with Fourier‐space time‐series phase‐shifting; (3) nonbrain structures removal with Brain Extraction Tool (BET) (Smith, 2002); (4) spatial smoothing with a Gaussian kernel of FWHM 5 mm; (5) grand‐mean intensity normalization of the entire 4D dataset by a single multiplicative factor; and (6) high‐pass temporal filtering (Gaussian‐weighted least‐squares straight line fitting, with sigma = 50 sec).

Each participant's functional data were first registered to their high‐resolution structural image (7 df) and then registered to a standard space (MNI‐152 atlas, 12 df) using the FLIRT (FMRIB's Linear Image Registration Tool) (Jenkinson and Smith 2001; Jenkinson et al. 2002) and the FNIRT nonlinear registration (Andersson et al. 2007a,b).

The analysis of each participant's functional data was performed using FSL's implementation of the general linear model in FEAT (FMRI Expert Analysis Tool, Version 5.98, FMRIB Analysis Group, Oxford, U.K.). The model included motion parameters and a separate regressor for each condition: traits, facts, interoception, and exteroception. Each regressor derived from the convolution of the corresponding task design and a double gamma function (representing the hemodynamic response). Time‐series statistical analysis was conducted with FILM using a local autocorrelation correction (Woolrich et al. 2001).

For each participant, the functional data from three runs were then analyzed with a fixed‐effect model, which forces the random effects variance to zero in FLAME (FMRIB's Local Analysis of Mixed Effects) (Beckmann et al. 2003; Woolrich et al. 2004).

Finally, the data from all the participants were analyzed using a mixed‐effect model, FLAME (Beckmann et al. 2003; Woolrich et al. 2004). For the whole‐brain analysis, a threshold was applied to all statistical images, using cluster size probability to correct for multiple comparisons. Only voxels with values *Z *>* *2.3 and occurring in clusters with a corrected cluster size significance threshold of *P *=* *0.05 passed the threshold (Worsley, 2001).

Conjunction analyses for higher level contrasts were performed using the easythresh_conj script in FSL (Nichols 2007), with the whole brain as a mask, and using the same threshold described for the previous analyzes (*Z *>* *2.3, cluster size *P *=* *0.05) in order to identify regions commonly activated by the conditions (Price and Friston 1997). The algorithm (“the Minimum Statistic compared to the Conjunction Null) does not require different baselines for the comparisons; rather, it is valid for comparisons of different tasks with the same baseline (Nichols et al. 2005).

In addition, we also determined mean PE (parameter estimates) for each condition‐minus‐baseline in masks for ROIs in CMSs. The ROI masks consisted of spheres with 5‐mm radius and were centered on peaks of activation for the varied contrasts explored in the study (Table S7). For each ROI, PE for each condition minus baseline in whole‐brain analysis were determined using FEATQUERY in FSL, and mean PE and SEM (standard error mean) PE were calculated. We note that the ROI analysis was not performed for inference purposes; it was rather performed to help visualize the differences of activation in CMSs across the conditions.

Response time data were compared across conditions using repeated measures ANOVA; and memory estimates were analyzed using paired *t*‐test. For both analyses, PASW Statistics 18.0 (SPSS, Quarry Bay, Hong Kong) was used.

## Results

### Behavioral data

#### Response times

Autobiographical self questions were answered faster (M* *=* *1.43 SEM = 0.04 sec) than core self questions (M* *=* *1.70, SEM = 0.07 sec), *F* (1, 18) = 31.46, *P *<* *0.0001. In addition, response times were shorter for interoception (M = 1.57, SEM = 0.06 sec) than exteroception (M* *=* *1.84, SEM = 0.07 sec), *F* (1,18) = 55.69, *P* < 0.0001.

#### Memory estimates

There was no statistically significant difference between traits and facts in relation to participants’ estimates of the number of memories required to answer the questions (facts: M* *=* *3.21, SEM = 0.371; traits: M* *=* *3.0; SEM = 0.286), *t* (18) = −0.001, *P* > 0.999. In addition, participants’ estimates of the number of memory episodes elicited by the questions were greater for interoception (M* *=* *2.32, SEM = 0.306) than for exteroception (M* *=* *1.68; SEM = 0.254), *t* (18) = 2.72, *P* < 0.014.

### Functional imaging data

#### Self versus one‐back baseline

A conjunction analysis of the higher level analysis results for each condition compared with baseline revealed that all four conditions overlapped in terms of activity in the following regions: bilaterally in the ventromedial prefrontal cortex, medial frontal gyrus, inferior PMC (comprising inferior precuneus, and the posterior cingulate and retrosplenial cortices), cuneus, inferior frontal gyrus/anterior insula, and cerebellum; and in the left superior and middle prefrontal gyri, middle and superior temporal gyri, angular gyrus, lateral occipital gyrus, hippocampus, and amygdala (Fig. S1).

### Autobiographical self versus core self

#### Autobiographical self > core self (interoception and exteroception)

**Figure 1:**
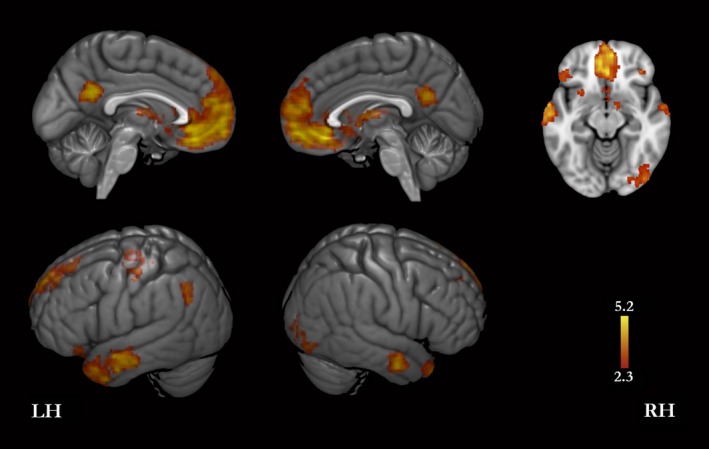
Facts and traits compared with core self conditions. These are results from a conjunction analysis for the following contrasts: facts > (interoception + exteroception), and traits > (interoception + exteroception). Thus, these brain regions showed greater signal for autobiographical self questions than for core self questions. LH, left hemisphere; RH, right hemisphere.

A conjunction analysis of the contrasts facts > core self and traits > core self showed that both facts and traits yielded higher level of activity than that generated for core self in the following regions: bilaterally, orbitofrontal cortex, MPFC, ACC, inferior PMC (comprising the inferior precuneus and the most superior part of the posterior cingulate cortex), middle temporal gyrus, temporal pole, thalamus, caudate, putamen, accumbens; superior parts of the left precentral and postcentral gyri; in the left superior parietal lobule, angular gyrus and anterior insula; and in the right amygdala (Fig. 1, Table 1). Results relative to the same comparison obtained by a regular subtraction analysis are presented as supplementary material (Fig. S2 and Table S1).

**Table 1:** Activation peaks for the conjunction of the following contrasts: facts > coreself (interoception + exteroception) and traits > coreself (interoception + exteroception). Thus, the peaks refer to brain regions showing greater signal for autobiographical self questions than for core self questions. Coordinates are in the MNI‐152 standard space

Structure	H	*x*	*y*	*z*	*Z*
Medial prefrontal cortex/anterior cingulate cortex	L	−4	36	−10	4.83
	R	2	32	−10	4.97
Posterior cingulate cortex/precuneus	L	−4	−52	26	4.78
	R	10	−52	22	4.46
Inferior frontal gyrus/orbitofrontal cortex	L	−46	26	−10	3.17
	R	38	34	−12	3.58
Precentral gyrus/postcentral gyrus	L	−48	−22	62	3.71
Temporal pole	L	−52	12	−28	4.02
	R	40	24	−36	4.02
Middle temporal gyrus	L	−62	−12	−14	4.83
	R	62	−6	−22	4.43
Lateral occipital cortex	L	34	−90	−8	4.06
Superior parietal lobule/angular gyrus	L	−50	−66	36	3.51
Insula	L	−28	12	−12	3.35
Caudate/putamen/accumbens	L	−6	14	−6	3.25
	R	4	14	−8	3.78
Thalamus	L	−2	−6	10	3.39
	R	4	−2	6	3.29
Amygdala	R	12	−6	14	2.75

H, hemisphere; L, left; R, right; *Z*,* Z*‐score.

The results regarding the comparison between facts and core self are presented in supplementary material (Fig. S3 and Table S2); likewise for the comparison between traits and core self (Fig. S4 and Table S3).

#### Core self > autobiographical self (traits and facts)

**Figure 2:**
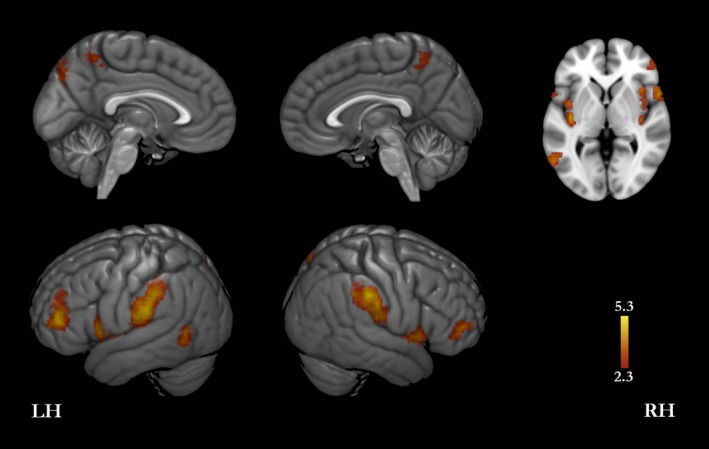
Interoception and exteroception compared with autobiographical self conditions. The images derive from a conjunction analysis for the following contrasts: interoception > (facts + traits), and exteroception > (facts + traits). Thus, these brain regions showed greater signal for core self questions than for autobiographical self questions. LH, left hemisphere; RH, right hemisphere.

A conjunction analysis of the contrasts interoception > autobiographical self and exteroception > autobiographical self revealed that compared with autobiographical self both interoception and exteroception were associated with higher level of activity bilaterally in the most superior and anterior part of the PMC, the inferior and middle frontal gyri, inferior part of the precentral gyrus, supramarginal gyri, and insula (including bilaterally the posterior insula, and the right anterior insula); in the left most superior and posterior part of the PMC, superior parietal lobule, and EBA (extrastriate body area; here as in the rest of this publication, the location of EBA is based on the coordinates published in Downing et al. 2001) (Fig. 2, Table 2). Results relative to the same comparison obtained by a regular subtraction analysis are presented as supplementary material (Fig. S5 and Table S4).

**Table 2:** Activation peaks for a conjunction analysis for the following contrasts: exteroception > autobiographical self (traits + facts) and interoception > autobiographical self (traits + facts). Thus, the peaks refer to brain regions showing greater signal for core self questions than for autobiographical self questions. Coordinates are in the MNI‐152 standard space

Structure	H	*x*	*y*	*z*	*Z*
Precuneus	L	−4	−52	58	3.11
	R	4	−54	58	3.40
Inferior frontal gyrus/middle frontal gyrus	L	−38	38	16	4.84
	R	50	48	8	3.59
Superior frontal gyrus/precentral gyrus	L	−56	10	6	4.27
	R	56	10	4	4.39
Supramarginal gyrus	L	−56	−28	32	4.78
	R	56	−24	28	4.64
Middle temporal gyrus/lateral occipital gyrus	L	−58	−62	6	4.13
Superior parietal lobule	L	−32	−52	40	3.96
Insula	L	−40	−12	−4	5.34
	R	40	−4	−8	4.45

H, hemisphere; L, left; R, right; *Z*,* Z*‐score.

The results obtained by the comparison between interoception and autobiographical self are presented as supplementary material (Fig. S6 and Table S5); likewise for the comparison between exteroception and autobiographical self (Fig. S7 and Table S6).

### Facts versus traits

**Figure 3:**
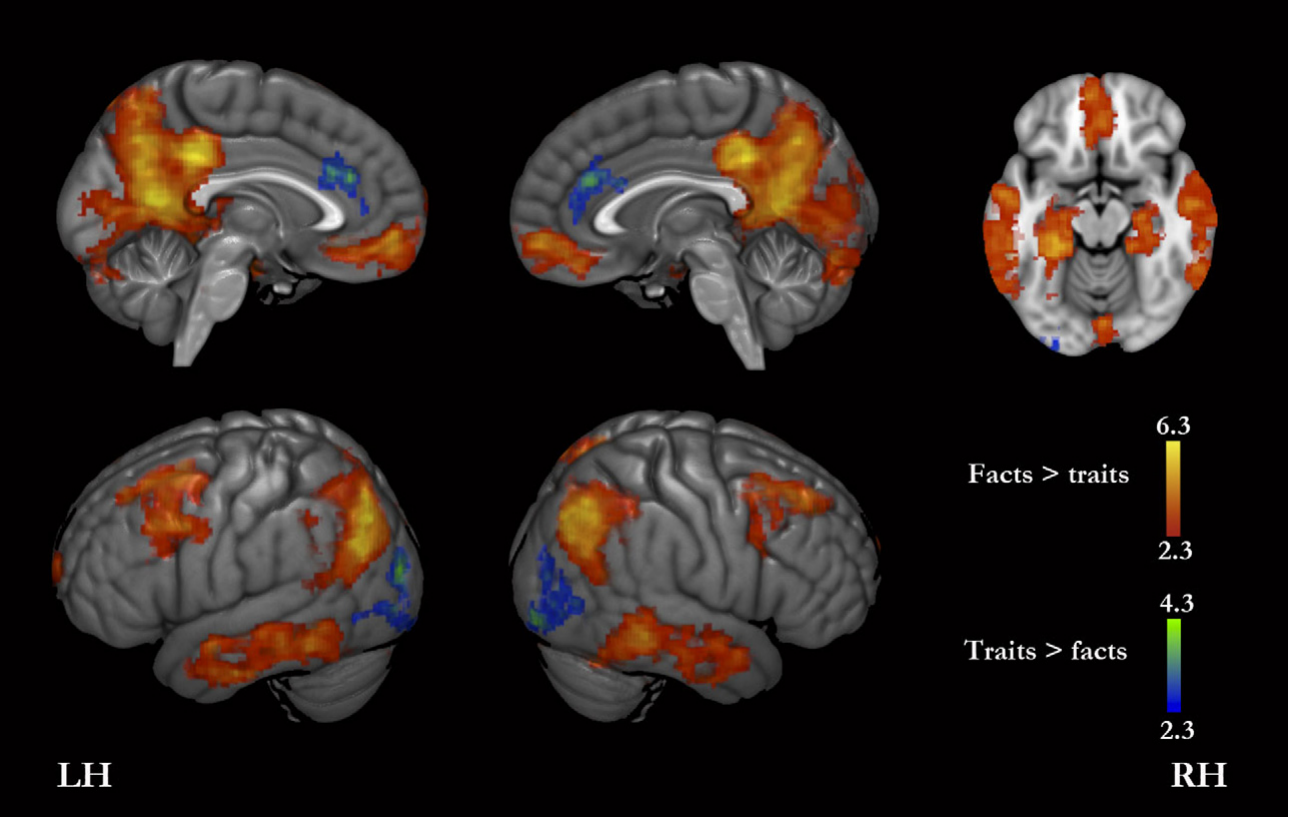
Facts versus traits. The red‐yellow color scale shows brain regions with significantly greater signal for facts than for traits. The blue‐green scale shows brain regions with greater signal for questions targeting traits than for those targeting facts. LH, left hemisphere; RH, right hemisphere.

Facts compared with traits showed greater activity bilaterally in the MPFC, PMC (comprising all the PMC except for the most superior and anterior part); superior and middle frontal gyri, including clusters adjacent to the precentral sulcus (premotor cortices); supramarginal and angular gyri, superior parietal lobule, middle and inferior temporal gyri, amygdala, hippocampus and hippocampal formation, fusiform gyrus, and cerebellar cortex, and in the left frontal pole, and pons (Fig. 3, Table 3).

**Table 3:** Activation peaks for facts versus traits. Coordinates are in the MNI‐152 standard space

Structure	H	*x*	*y*	*z*	*Z*
*Facts > traits*					
Medial prefrontal cortex	R/L	0	56	−4	5.29
	L	−6	52	−8	5.14
	R	6	46	−10	4.44
Posterior cingulate cortex/retrosplenial cortex/precuneus	L	−4	−60	14	6.34
	L/R	0	−60	14	6.29
Frontal pole	L	−16	64	8	3.5
Superior/middle frontal gyri	L	−22	20	48	5.13
	R	26	22	48	5.44
Supramarginal gyrus/angular gyrus/superior parietal lobule	L	−46	−68	32	6.33
	R	42	−74	38	6.02
Middle/inferior temporal gyrus	L	−58	−48	−8	6.08
	R	62	−44	−8	5.69
Amygdala	L	−22	−6	−12	3.10
	R	22	−8	−20	2.82
Hippocampus/hippocampal formation	L	−20	−18	−20	4.42
	R	24	−20	−16	4.37
Fusiform gyrus	L	−24	−38	−14	5.32
	R	30	−32	−18	4.09
Cerebellar cortex	L	−12	−74	−28	3.93
	R	48	−64	−24	4.03
Pons	L	−10	−30	−32	3.76
*Traits > facts*					
Lateral occipital gyrus	L	−34	−94	16	4.39
	R	40	−92	−8	3.9
Angular gyrus	R	54	−58	2	2.9
Anterior cingulate cortex/posterior medial frontal gyrus	L/R	0	34	24	4.23

H, hemisphere; L, left; R, right; *Z*,* Z*‐score.

On the other hand, the reverse contrast showed that the level of activity bilaterally in the lateral occipital gyrus, posterior ACC (midcingulate cortex) and medial frontal gyrus, and right angular gyrus was higher for traits than for facts (Fig. 3, Table 3).

### Interoception versus exteroception

**Figure 4:**
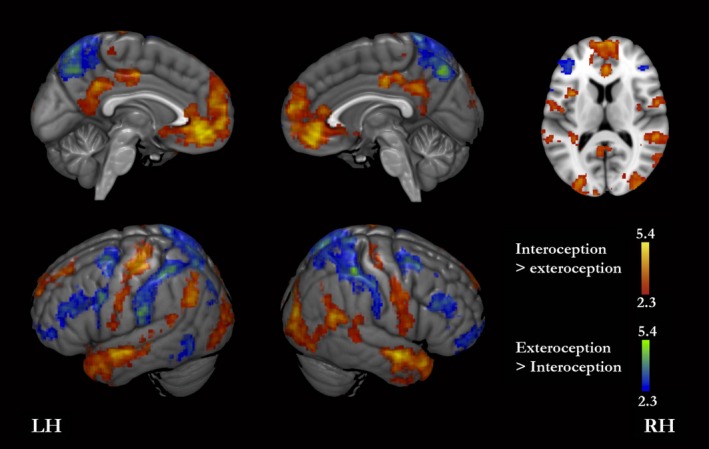
Interoception versus exteroception. The red‐yellow color scale shows brain regions with significantly greater signal for interoception than for exteroception. The blue‐green scale shows brain regions with greater signal for questions regarding exteroception than for those regarding interoception. LH, left hemisphere; RH, right hemisphere.

Interoception compared with exteroception yielded greater activity bilaterally in MPFC, ACC, paracentral gyrus, inferior PMC (comprising the posterior cingulate cortex, and inferior precuneus), precentral and postcentral gyri, superior and medial temporal gyri, lateral occipital gyrus, angular gyrus, and insula (clusters bilaterally in the anterior insula, and in the left posterior insula); in the left SMG (supramarginal gyrus), superior parietal lobule, hippocampus, caudate/accumbens, and EBA (Fig. 4, Table 4).

**Table 4:** Activation peaks for interoception versus exteroception. Coordinates are in the MNI‐152 standard space

Structure	H	*x*	*y*	*z*	*Z*
*Interoception > exteroception*					
Medial prefrontal cortex	L	−4	48	−10	5.4
	R	6	60	16	4.84
Anterior cingulate cortex	L	−2	42	−8	5.2
	R	2	40	−8	5.01
Paracentral gyrus	L	−4	−34	64	2.84
	R	10	−34	70	3.01
Posterior cingulate cortex	L	−4	−18	40	4.42
	R	2	−18	40	4.38
Precuneus	L	−2	−48	36	4.5
	R	2	−48	32	4.02
Superior frontal gyrus	L	−18	46	48	3.81
Precentral gyrus/postcentral gyrus	L	−36	−24	70	4.39
	R	64	4	18	3.91
Superior temporal gyrus/medial temporal gyrus	L	−56	−6	−12	5.32
	R	62	−4	−10	5.42
Temporal pole	L	−54	10	−24	4.21
	R	50	18	−24	4.95
Lateral occipital gyrus	L	−40	−84	0	4.28
	R	34	−90	18	4.76
Middle temporal gyrus/lateral occipital gyrus	R	54	−68	6	2.87
Supramarginal gyrus/angular gyrus	L	−44	−38	22	4.23
	R	62	−50	22	4.7
Superior parietal lobule	L	−50	−68	36	4.23
Hippocampus	L	−26	−14	−14	3.41
Caudate/accumbens	L	−12	6	−8	3.78
Insula	L	−40	8	−8	4.03
	R	40	10	−8	3.04
*Exteroception > interoception*					
Precuneus	L	−10	−70	52	4.99
	R	4	−68	48	5.09
Frontal pole/orbitofrontal	L	30	46	−16	4.21
	R	−22	54	−18	2.85
Superior frontal gyrus/Middle frontal gyrus	L	−26	6	64	4.84
	R	30	6	60	5.06
Precentral gyrus/inferior frontal gyrus/middle frontal gyrus	L	−52	6	36	4.55
Supramarginal gyrus/superior parietal lobule	L	−62	−26	22	5.17
	R	46	−42	60	5.28
Middle temporal gyrus/lateral occipital gyrus	L	−52	−66	−2	4.17

H, hemisphere; L, left; R, right; *Z*,* Z*‐score.

On the other hand, the reverse contrast (exteroception > interoception) yielded greater activity bilaterally in superior the PMC (comprising the most superior part of the precuneus, extending from its anterior limit to its posterior limit), frontal pole and orbitofrontal cortex; in the middle and inferior frontal gyri adjacently to the inferior frontal sulcus; in the superior and middle frontal gyrus adjacently to the precentral sulcus (premotor cortices); in the SMG, and superior parietal lobule; in the left inferior frontal gyrus adjacently to the precentral sulcus (premotor cortex), and EBA (Fig. 4, Table 4).

#### Cortical midline structures

In order to summarize and to provide an additional way to visualize the level of activity in CMSs across conditions, we calculated mean PE for each condition‐minus‐baseline in ROI masks for the MPFC and PMC. The ROIs were determined by activation peaks yielded in these regions for the contrasts described above (summarized in Table S7).

#### Medial prefrontal cortex and anterior cingulate cortex

**Figure 5:**
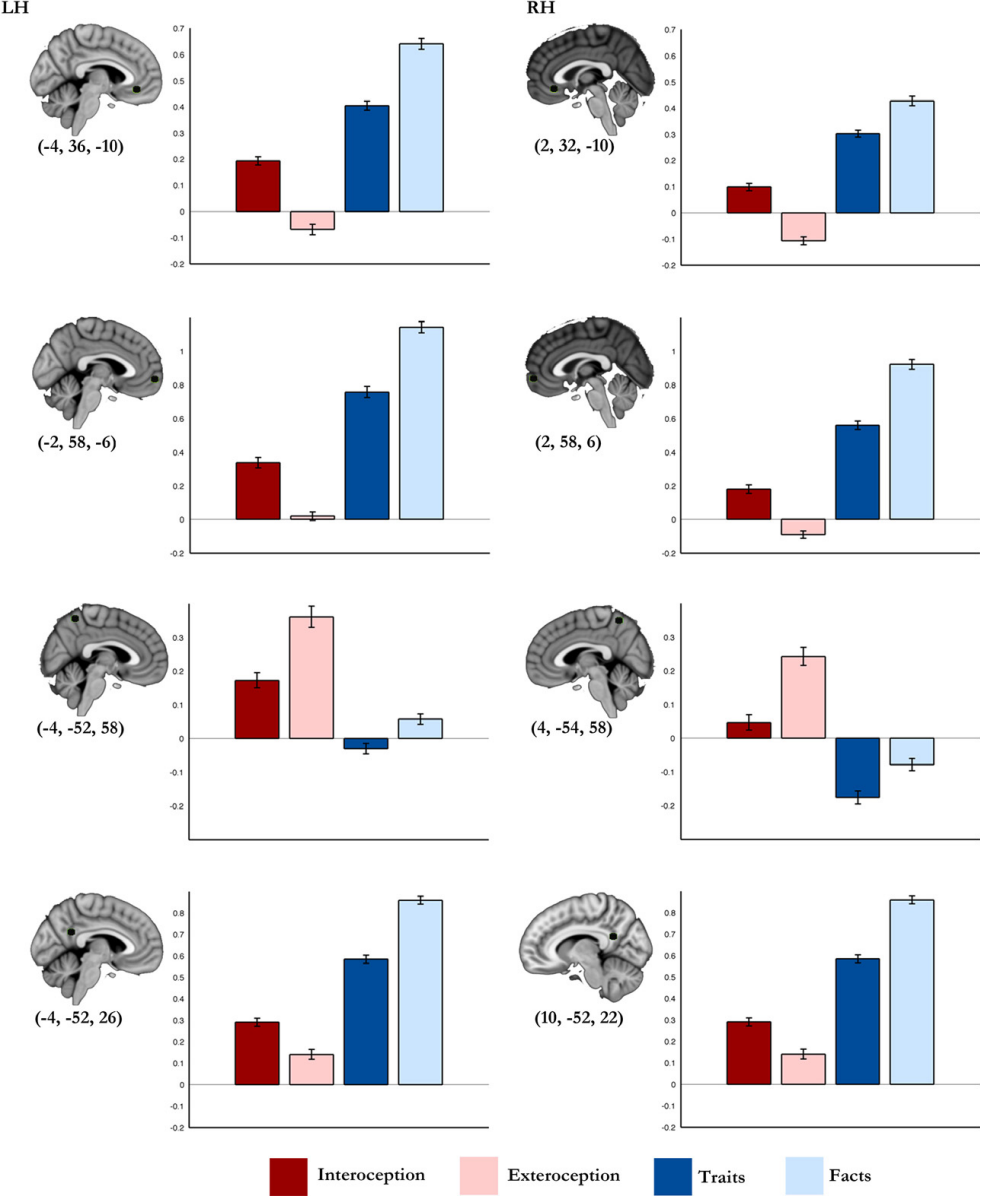
Parameter estimates for each condition in cortical midline structures. Regions of interests consisted of spheres of 5 mm radius centered on activation peaks in the medial prefrontal cortex and in the posteromedial cortex from the conjunction analyses. MNI coordinates (*x*,* y*,* z*) in parentheses; error bars represent standard error mean.

The MPFC (along with adjacent subgenual and pregenual ACC) yielded greater activity for autobiographical self than for core self (Figs. 1, 5, S2). In addition, the MPFC showed greater activity for facts than for traits (Figs. 3, 5); and for interoception than for exteroception (Figs. 4, 5).

We note, however, that a posterior and rostral part of the ACC (the midcingulate cortex), showed greater activity for traits than for any other conditions (Figs. 3, S4).

#### Posteromedial cortex

The most superior and anterior PMC showed greater activity for core self conditions than for autobiographical self conditions (Figs. 2, 5). Furthermore, the comparison between conditions revealed that the superior PMC yielded greater activity for exteroception than for interoception (Figs. 4, 5, S9); likewise, it showed greater activity for facts than for traits (Figs. 3, 5, S9).

On the other hand, regions within the inferior PMC, specifically the inferior precuneus, and the posterior cingulate cortex, showed greater activity for autobiographical self conditions than for core self conditions (Figs. 1, 5, S8). Moreover, the level of activity in the inferior PMC was greater for facts than for traits (Figs. 3, 5, S8, S9). We note, however, an ROI located in the posterior cingulate cortex that appeared to show greater activity for traits than for facts (Fig. S8) but this result did not reach significance in the GLM‐contrast (Fig. 3). In addition, the level of activity in regions within the inferior PMC (namely in the inferior precuneus, the retrosplenial cortex, and the posterior cingulate cortex) was greater for interoception than for exteroception (Figs. 4, 5, S8, S9).

## Discussion

The results above confirm that self‐related mental states are not unitary or monolithic, but instead they vary in terms of neural and behavioral structures. Moreover, the data reveal similarities as well as differences across those states.

Both autobiographical self and core self questions elicited activity in the midline and ICs, as well as in lateral frontal, temporal and parietal cortices, hippocampus and amygdala. This anatomical and functional overlap includes regions related to memory (e.g., hippocampus) and to body representations (e.g., somatosensory, premotor, and motor cortices). It thus supports the hypothesis that both autobiographical self and core self states are, to a certain extent, associated with memory‐related and body‐related processing.

The data also revealed differences across the conditions. In the sections below, we discuss the differences relative to activity in ROI in this study; in addition, we advance an interpretation for the role of CMSs and ICs in processing self‐related information.

### Memory‐related brain regions and self‐relates mental states

Memory‐related regions were more active for autobiographical self than for core self, as hypothesized. The anterior temporal cortices, which have been implicated in the retrieval of semantic knowledge regarding self (Kjaer et al. 2002; Lou et al. 2004) and others (Olson et al. 2007; Von Der Heide 2013), showed greater activity for autobiographical self conditions than for core self conditions.

In addition, the hippocampus showed greater activity for facts than for traits. Given the established role of the hippocampus in memory retrieval (see Cohen & Eichenbaum, 1993), this suggests that mental states evoked by questions about facts elicited greater amount of memory retrieval than those evoked by questions about traits. This difference may be explained by the likely possibility that individuals hold a greater number of memory representations for facts than for traits. Facts are particularly relevant to one's identity and daily life, and the number of daily events and experiences related to facts is large. On the other hand, not all traits are relevant to one's identity and daily life, and the number of daily events and experiences related to traits in one's life is presumably more limited than that related to facts.

The hippocampus also showed greater level of activity for interoception than exteroception, suggesting that interoceptive sensations trigger greater amount of memory retrieval than exteroceptive sensations. This suggestion is supported by participants’ estimates of the amount of memory retrieval elicited by the questions, which were greater for interoception than for exteroception, and is compatible with the notion that interoception are important cues for memory retrieval (Hirsh 1974).

### Body‐related brain regions and self‐related mental states

Both core self conditions yielded greater activity in the SMG than autobiographical self conditions. Because the SMG is reciprocally connected with the insular, somatosensory and premotor cortices (e.g., Andersen et al., 1990), and has been involved in processing varied body sensations (Nour et al. 2000; Committeri et al. 2007; Kuhtz‐Buschbeck et al. 2007), this finding suggests that body‐related regions are more active for core self states than for autobiographical self states. This appears to be further supported by the comparison between each core self condition with the autobiographical self conditions. For example, compared with the autobiographical self conditions, interoception was associated with greater activity in the somatosensory cortices and motor cortices, and exteroception, with greater activity in the premotor and motor cortices.

The EBA was more active for core self than for autobiographical self. Given that the EBA has been shown to be preferentially activated by images of the human body and body parts (Downing et al. 2001), this finding suggests that core self states are associated with a greater amount of body‐related visual imagery than autobiographical self states.

Nonetheless, compared with core self questions, autobiographical self questions elicited a higher level of activity in varied regions involved in processing emotion‐related somatic representations, such as the anterior cingulate cortex (Vogt 2005; Vogt and Palomero‐Gallagher 2013) and the amygdala (Pessoa and Adolphs 2010). This suggests that autobiographical self mental states are associated with a greater extent of emotion‐related processing, probably having largely to do with emotional responses to the memories retrieved. Several prior findings are in line with this view. The subgenual ACC is involved in emotional processes related with memory retrieval such as recalling sad memories (reviewed in Vogt and Palomero‐Gallagher 2013). Also, the amygdala has been linked to the consolidation and retrieval of memories with high emotional content (Denkova et al. 2013).

In addition, the posterior and rostral ACC, sometimes designated as midcingulate cortex (Vogt and Palomero‐Gallagher 2013), was more active for traits than facts. This difference may relate to decision processes, given the association between the midcingulate cortex and decision‐making (Ridderinkhof 2004; Vogt and Palomero‐Gallagher 2013). Deciding whether a “trait” is self descriptive is probably less straightforward than deciding whether a “fact” is self descriptive (Keenan et al. 1992; Araujo et al. 2013).

### CMS and self‐related mental states

#### CMSs and the DMN (default mode network)

Our data appear to confirm the involvement of CMSs, particularly the MPFC and the PMC, along with other regions of the DMN (e.g., the lateral temporal cortex and angular gyrus), in self‐related mental states (Qin and Northoff 2011). Moreover, the level of activity in those DMN regions was greater for autobiographical self states than for core self states. This finding supports the notion that DMN regions are particularly engaged by states in which individuals temporarily disengage from what is happening in the external world or in their bodies, and focus on the retrieval, display and manipulation of internally generated representations (e.g., memories and related thoughts).

Intriguingly, the level of activity in the MPFC, inferior PMC, lateral temporal cortex and angular gyrus, was greater for interoception than for exteroception. This may relate to differences in memory retrieval between the conditions, but it may well suggest that, compared with autobiographical self mental states, DMN's involvement in core self states is more restricted when the focus is on exteroceptive sensations. Other studies seem to support this suggestion. It has been shown that DMN activity correlates negatively with activity in the somatosensory cortices (Fox et al. 2008) and in the auditory and visual cortices (Tian et al. 2007).

#### The MPFC

As noted above, the level of activity in the MPFC was greater for: (1) autobiographical self conditions than for core self conditions; (2) facts than for traits; and (3) interoception than for exteroception. Because there is substantial evidence that the MPFC assists the participation of emotion‐related somatic representations in decision (Bechara et al. 2000), and evaluative processes (D'Argembeau 2013), we believe that MPFC activity during self‐related mental states is commensurate with the extent of emotion‐related processing in those states.

The extent of emotion‐related processing in a given state is, in turn, probably commensurate with the number of elements that are being processed and are involved in inducing an emotional response. In our study, the mental states evoked by the conditions seem to involve predominantly processing of memories and body sensations. In addition, as noted before, memories include manifold elements pertaining to the events and experiences from which those memories derived, while body sensations, particularly those within the homeostatic range, are probably more limited in that regard. Accordingly, memories may have a greater potential of eliciting emotion‐related processing than body sensations, and this may well explain the difference of activity in the MPFC between core self and autobiographical self. Moreover, it may also explain the differences of activity in the MPFC between facts and traits because facts are associated with a greater amount of memory retrieval; likewise for the difference between interoception and exteroception, given that interoceptive questions seem to elicit greater amount of memory retrieval.

Findings from other studies support our proposal in relation to the MPFC. The level of activity in the MPFC has been shown to be commensurate with variables that, in all likelihood, correlate with the extent of emotional processing, namely: one's level of experience, familiarity or affective closeness with the stimuli (i.e., objects or people) that are processed in different studies. For instance, MPFC is more active when individuals process objects with which they are highly experienced than when they process objects with which they are not highly experienced (Lieberman et al. 2004). In addition, the MPFC's involvement in processing information about other people seems to be greater for affectively closer individuals (e.g., relatives) than for affectively more distant people (Ochsner et al. 2005; Zhu et al. 2007).

### The PMC and its sub‐regions

#### The superior PMC

The *most superior PMC* (i.e., superior precuneus) was more active for exteroception than for interoception and than for any of the autobiographical self conditions, suggesting that this region is particularly involved in processing exteroceptive body changes. Data from other studies support this conclusion. For instance, as mentioned before, the most superior PMC is highly connected with somatosensory cortices and premotor and motor cortices (Parvizi et al. 2006). It has also been shown that the activity in the superior PMC during experiences of admiration and compassion is greater when those feelings relate to another person's body actions (e.g., admiration for a person's performance at gymnastics or compassion for a person's physical pain caused by a broken leg) than when the feelings relate to another person's “psychological” state (e.g., admiration for a social virtue, such as generosity; and compassion for someone who grieves the death of a close one) (Immordino‐Yang et al. 2009).

We note, however, that in our study, *the most anterior and superior PMC* (i.e., superior precuneus adjacent to the ascending ramus of the cingulate sulcus) showed greater level of activity not only for exteroception but also for interoception, compared with the autobiographical self conditions. This finding suggests that the most anterior and superior PMC is involved in processing varied domains of body sensations, a suggestion that is compatible with findings from resting state functional connectivity. Specifically, it has been shown that this region is connected with the primary and secondary somatosensory cortices as well as with the ICs (Zhang et al. 2014).

#### The inferior PMC

The *most inferior PMC,* comprising predominantly the more posterior Posterior Cingulate Cortex (PCC), the retrosplenial cortex and the most inferior precuneus, appears to be active during all conditions. This may indicate that hubs in the PMC are of a relatively general purpose and can assist varied processes. This indication is supported by findings derived from other studies. For instance, it is known that the ventral precuneus is a central structure of the so‐called default network (Zhang et al. 2014), and that the retrosplenial cortex is involved in a wide range of cognitive functions, from memory retrieval, mind wandering, imagination and spatial navigation tasks (Vann et al. 2009).

A relatively *more dorsal region within the inferior PMC*, comprising the inferior precuneus and adjacent PCC, showed greater activity for autobiographical self than for core self. In a different study, a similarly located region of the PMC generated greater activity for pictures that participants considered more self‐relevant than for those they considered less self‐relevant (Sajonz et al. 2010). It is possible that activity in this region relates to memory retrieval given the presumable difference of memory retrieval across the conditions discussed above. In addition, it is known that the inferior precuneus holds a greater level of connectivity with the hippocampus than the superior precuneus (Zhang and Li 2012), and the inferior precuneus has also been implicated in retrieval of self‐related as well as nonself‐related memories (Cavanna 2006).

The *most anterior part of the PCC* seems to show greater level of activity for interoception than for exteroception. This is compatible with findings from anatomical connectivity studies showing that the cingulate cortex is connected with brainstem nuclei related to interoception (Cameron 2009).

### Insular cortices and self‐related mental states

Our data seem to confirm that ICs are involved in core and autobiographical self mental states, but that their involvement depends on the domain of self that is being processed in those states. More specifically, the most anterior insular cortex yielded greater activity for autobiographical self questions than for core self questions, and the most posterior insular cortex showed greater activity in the opposite direction. These findings are consistent with the proposal that activity in the more posterior insula relates predominantly to processing of somatic representations regarding “actual” body changes, whereas activity in the more anterior sectors of the insula is largely involved in processing of somatic representations related to emotions (see Craig 2002, 2009). It has also been shown that, in meditators, the posterior insula shows greater activity when they reflect on their body status than on their personality traits (Farb et al. 2007).

### Examining one's self varies in terms of response times

Answering core self questions took longer than answering autobiographical self questions. This suggests that the representations that are needed to answer core self questions are less readily available than those accessed to answer autobiographical self questions. To answer autobiographical self questions, it is necessary to access memory representations for relatively stable aspects of one's biography, namely for personality traits and biographical facts. Even though access to memories of a remote and insignificant life events may be relatively laborious, access to the memories related to autobiographical self questions was probably relatively effortless because individuals tend to hold summary representations for the information targeted by those questions (i.e., facts, and traits) given its relevance to one's identity and personality (Klein 2012).

On the other hand, to answer core self questions, individuals needed to access transitory maps of one's ongoing body status. Moreover, the mapping of body changes occurs in different levels of nervous system, and in part is processed in unconscious manner. Body signals within the homeostatic range are less readily accessible to consciousness than body signals that deviate from homeostasis, as shown in heartbeat detection tasks (Khalsa et al. 2009). We note that, in our study, the participants answered “no” to most of the questions targeting negative body sensations for interoception (e.g., hunger) or exteroception (e.g., pressure exerted on one's arm).

## Conclusion

Our data support the notion that neural correlates of self states vary depending on the information domain that is being considered during those states. It seems possible to distinguish the different self states in terms of activity in memory‐related regions and body‐related regions, as well to activity in regions that have been shown to be associated with self processes, namely the prefrontal and posteromedial cortices, and the ICs. Nonetheless, the data revealed a certain degree of overlap across self states, which emphasizes that the complexity of mental states elicited by self‐related questions. Those states they do not recruit solely regions that are required by the process of answering those questions. States elicited by questions focusing on one's current body status recruit somatosensory regions, as well as memory‐related regions possibly because those questions trigger memory retrieval. States elicited by questions focusing on historical aspects of oneself recruit memory‐related regions as well as body‐related regions possibly because the memories retrieved during those states evoke emotional responses.

## Conflict of Interest

None declared.

## Supporting information


**Figure S1**. Experimental conditions versus baseline.
**Figure S2**. Facts and traits compared with core self conditions.
**Figure S3**. Facts minus core self (interoception and exteroception).
**Figure S4**. Traits minus core self (interoception and exteroception).
**Figure S5**. Interoception and exteroception compared with autobiographical self conditions.
**Figure S6**. Interoception minus autobiographical self (facts and traits).
**Figure S7**. Exteroception minus autobiographical self (facts and traits).
**Figure S8**. Parameter estimates for each condition in CMSs.
**Figure S9**. Parameter estimates for each condition in CMSs.
**Table S1.** Activation peaks for the contrast autobiographical self (facts + traits) minus core self (interoception + exteroception).
**Table S2.** Activation peaks for the contrast facts minus core self (interoception + exteroception).
**Table S3.** Activation peaks for the contrast traits minus core self (interoception + exteroception).
**Table S4.** Activation peaks for the contrast core self (interoception + exteroception) minus autobiographical self (facts + traits).
**Table S5**. Activation peaks for the contrast interoception minus autobiographical self (traits and facts).
**Table S6.** Activation peaks for the contrast exteroception minus autobiographical self (traits and facts).
**Table S7.** Activation peaks (and the corresponding contrasts) used for ROI masks of CMSs (H: hemisphere; L, left; R, right; Z: Z‐score).
