# Supplementary material for: Neural correlates of different self domains
Source: Brain Behav. 2015 Oct 21;5(12):e00409. doi: 10.1002/brb3.409 (PMC4714646; doi:10.1002/brb3.409)
Supplement: Supplementary file 1 — Figure S1. Experimental conditions versus baseline. Figure S2. Facts and traits compared with core self conditions. Figure S3. Facts minus core self (interoception and exteroception). Figure S4. Traits minus core self (interoception and exteroception). Figure S5. Interoception and exteroception compared with autobiographical self conditions. Figure S6. Interoception minus autobiographical self (facts and traits). Figure S7. Exteroception minus autobiographical self (facts and traits). Figure S8. Parameter estimates for each condition in CMSs. Figure S9. Parameter estimates for each condition in CMSs. Table S1. Activation peaks for the contrast autobiographical self (facts + traits) minus core self (interoception + exteroception). Table S2. Activation peaks for the contrast facts minus core self (interoception + exteroception). Table S3. Activation peaks for the contrast traits minus core self (interoception + exteroception). Table S4. Activation peaks for the contrast core self (interoception + exteroception) minus autobiographical self (facts + traits). Table S5. Activation peaks for the contrast interoception minus autobiographical self (traits and facts). Table S6. Activation peaks for the contrast exteroception minus autobiographical self (traits and facts). Table S7. Activation peaks (and the corresponding contrasts) used for ROI masks of CMSs (H: hemisphere; L, left; R, right; Z: Z‐score). [file BRB3-5-e00409-s001.docx]

**
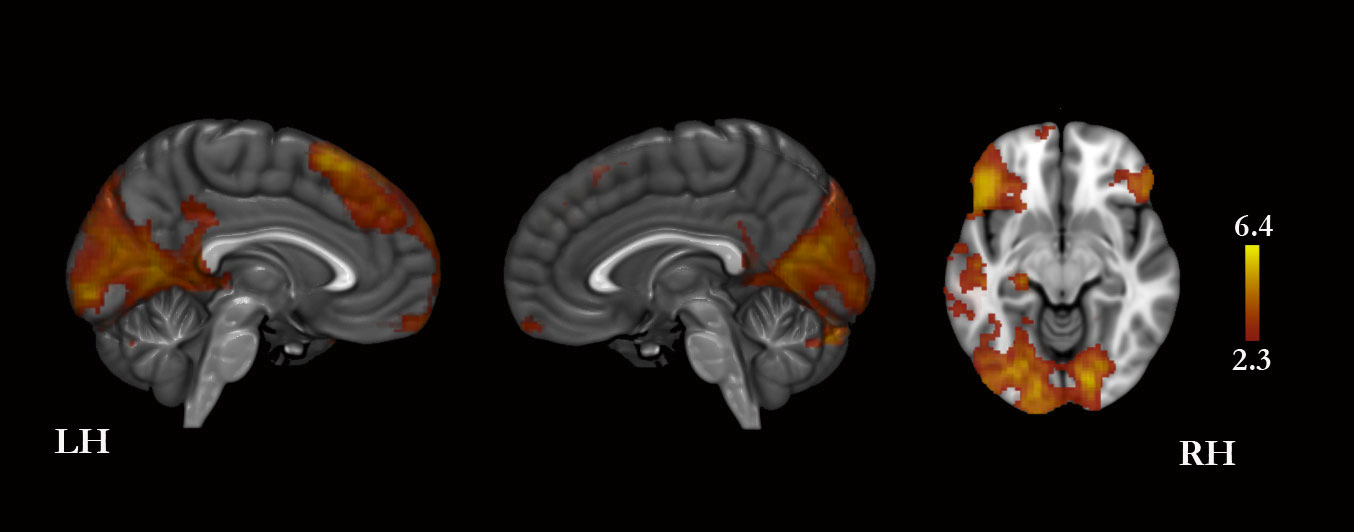
**

**Figure S1**. Experimental conditions versus baseline. The images derive from a conjunction analysis, and show brain regions with significantly greater signal during all of the self conditions relative to the baseline n-back task. (LH: left hemisphere; RH: right hemisphere).

**Autobiographical self > core self (interoception and exteroception)**

Altogether, autobiographical self conditions (facts and traits considered together) compared with core self conditions (interoception and exteroception considered together) showed greater level of activity bilaterally in the MPFC, ACC, paracentral gyrus, inferior PMC (comprising the posterior cingulate cortex, retrosplenial cortex and inferior precuneus), superior, middle and inferior frontal gyri, orbitofrontal cortex, temporal pole, middle and inferior temporal gyri, superior parietal lobule and angular gyrus, lateral occipital cortex, insula, caudate, putamen, accumbens, thalamus, and hippocampus; and in the right amygdala (Figure S2, Table S1).

**
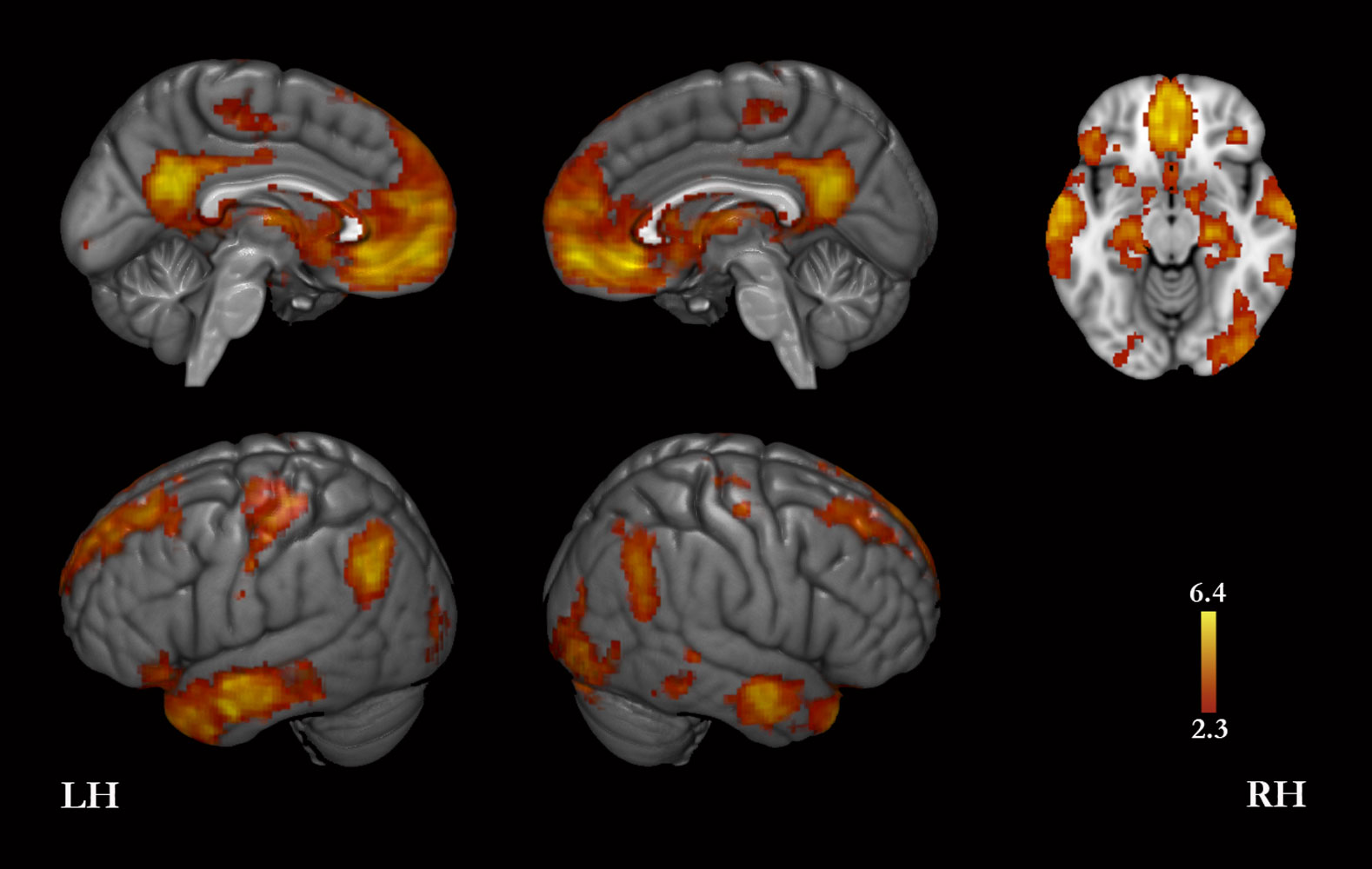
**

**Figure S2**. Facts and traits compared with core-self conditions. The images show brain regions with significantly greater signal during autobiographical self (facts + traits) compared with core self (interoception + exteroception). (LH: left hemisphere; RH: right hemisphere).

Facts compared with core self (interoception and exteroception considered together) yielded greater activity bilaterally in the orbitofrontal cortex, MPFC, anterior cingulate cortex (ACC), paracentral gyrus, inferior PMC (i.e., the posterior cingulate cortex, retrosplenial cortex, and approximately inferior half of the precuneus); middle and superior frontal gyri adjacently to the superior frontal sulcus; anterior part of the inferior frontal gyrus; superior parts of the precentral and postcentral gyri; superior parietal lobule, angular and lateral occipital gyri, middle temporal gyrus, temporal pole, thalamus, caudate, putamen, accumbens, hippocampus, and amygdala; in the left anterior insula; and in the right inferior temporal gyrus (Figure S3, and Table S2).

**
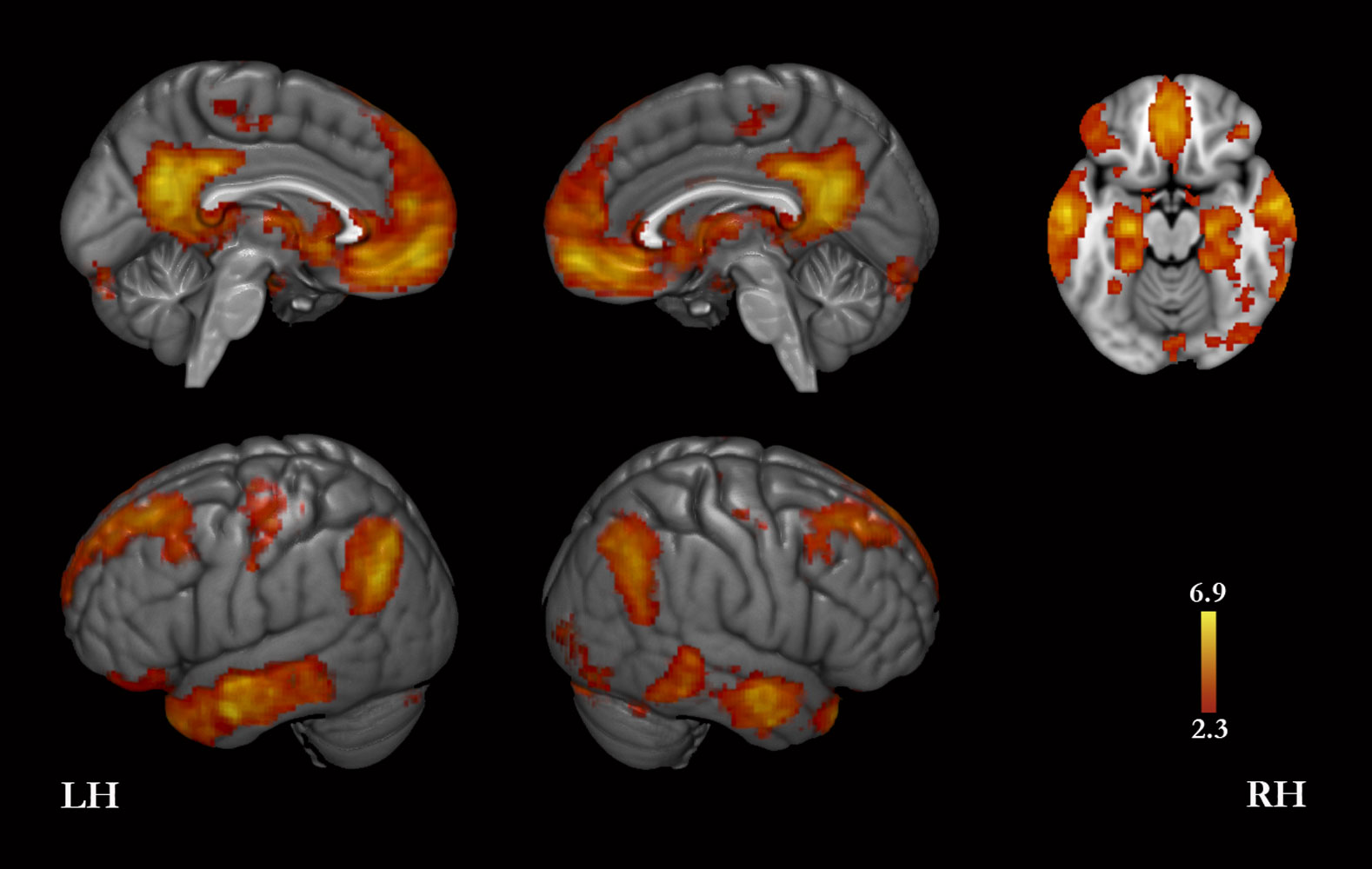
**

**Figure S3**. Facts minus core self (interoception and exteroception). The images show brain regions with significantly greater signal during facts compared with core self conditions. (LH: left hemisphere; RH: right hemisphere).

Traits compared with core self (interoception and exteroception considered together) showed greater level of activity bilaterally in the orbitofrontal cortex, MPFC, ACC, paracentral gyrus, inferior PMC (comprising the inferior precuneus and the most superior part of the posterior cingulate cortex); superior parts of the precentral and postcentral gyri; middle temporal gyrus, temporal pole, anterior insula, caudate, putamen, accumbens and thalamus; in the left superior parietal lobule and angular gyrus; and in the right amygdala and hippocampus (Figure S4, and Table S3).


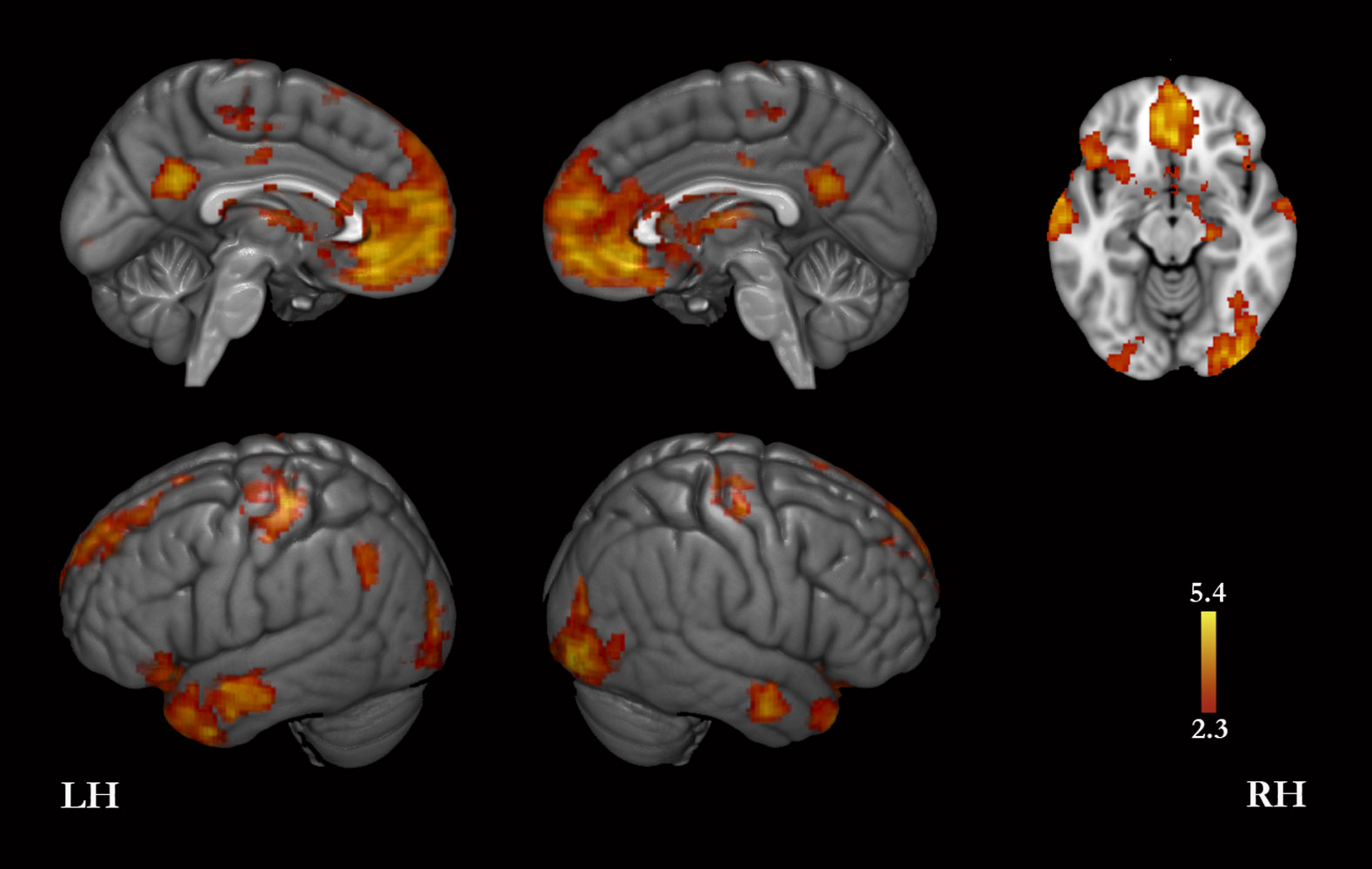


**Figure S4**. Traits minus core self (interoception and exteroception). The images show brain regions with significantly greater signal during traits compared with core self conditions. (LH: left hemisphere; RH: right hemisphere).

**Core self > autobiographical self (facts and traits)**

Altogether, core self (interoception and exteroception considered together) compared with autobiographical self (facts and traits considered together) generated greater activity bilaterally in the orbitofrontal cortex, the most superior PMC (i.e., the most superior precuneus), superior, middle and inferior frontal gyri, the precentral gyrus, and the insula; and in the left extrastriate body area (Figure S5, Table S4).


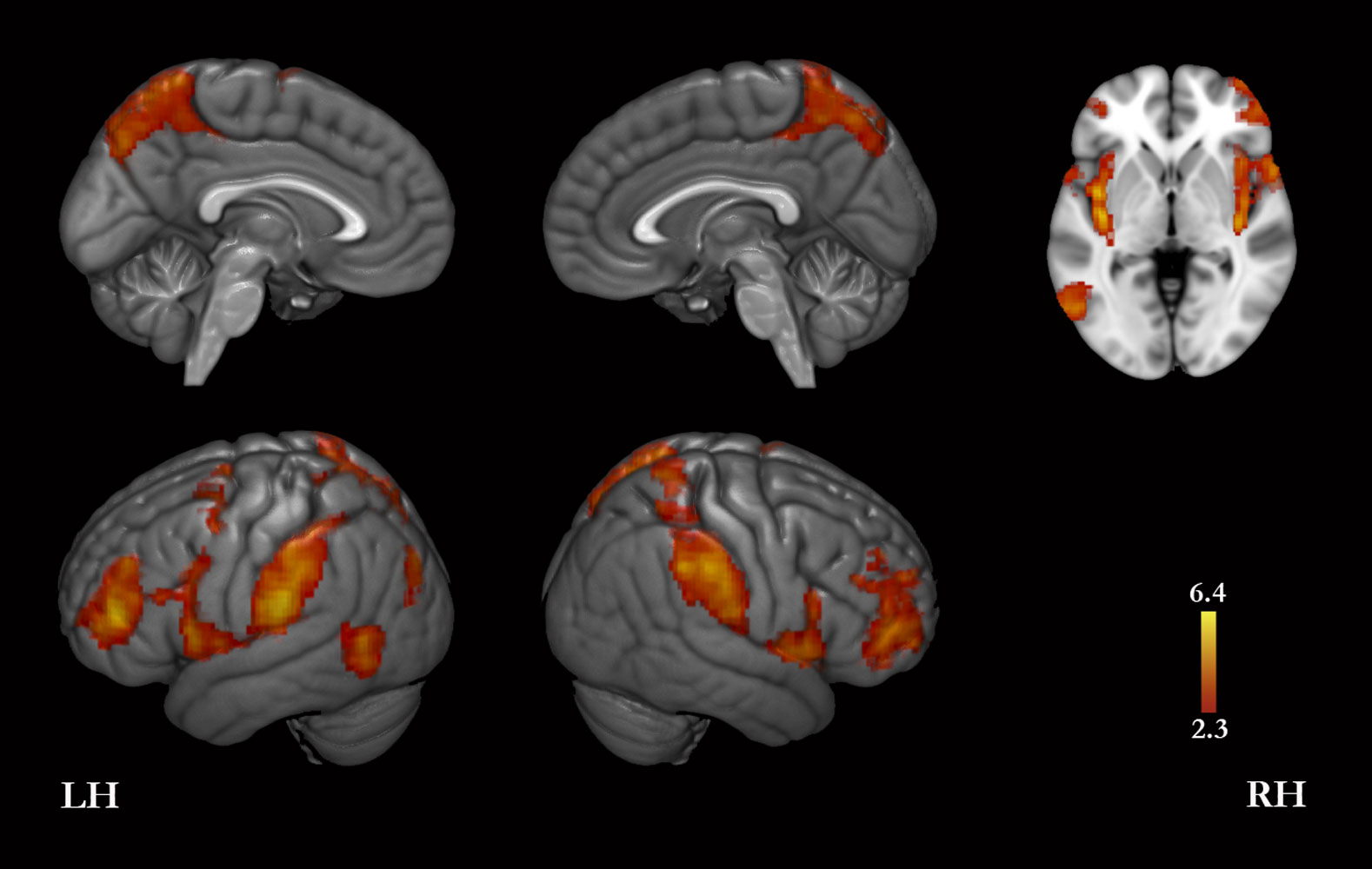


**Figure S5**. Interoception and exteroception compared with autobiographical-self conditions. The images show brain regions with significantly greater signal for core self (interoception + exteroception) than for autobiographical self (facts + traits). (LH: left hemisphere; RH: right hemisphere).

Interoception compared with the autobiographical self conditions (traits and facts considered together) was associated with greater activity bilaterally in the most superior and anterior PMC (i.e., comprising a cluster in the superior precuneus, adjacent to the ascending ramus of the cingulate sulcus), middle and inferior frontal gyri, inferior part of precentral gyrus, inferior part of the postcentral gyrus, supramarginal gyrus; middle temporal gyrus and adjacent lateral occipital cortex, and insula (including both anterior and posterior insula); in the left most superior and posterior part of the PMC (i.e., comprising a cluster in the superior precuneus adjacent to the occipitoparietal sulcus), and in the left extrastriate body area (here, as in the rest of this publication, the location of extrastriate body area is based on the coordinates published in (Downing, Jiang, Shuman, & Kanwisher, 2001); and in the right superior and inferior temporal gyri (Figure S6, Table S5).


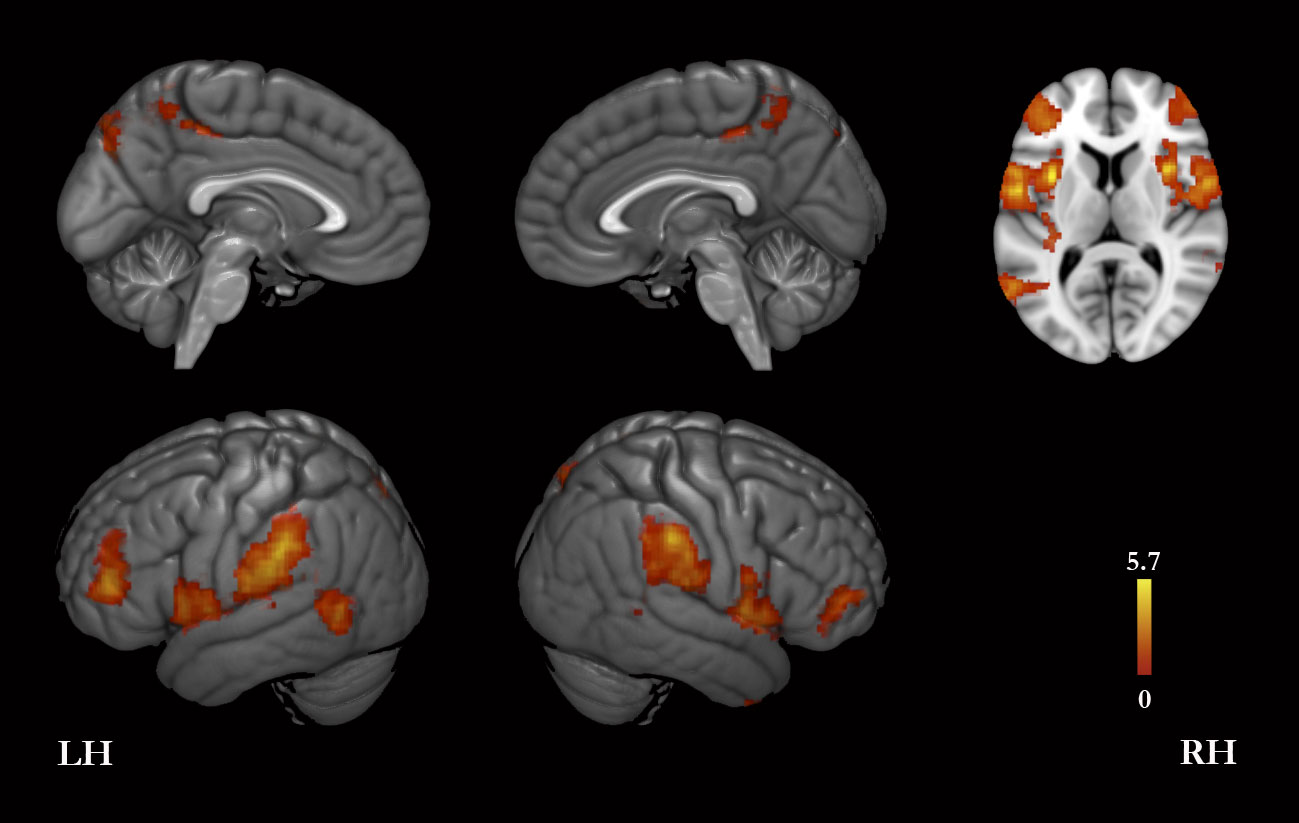


**Figure S6**. Interoception minus autobiographical self (facts and traits). The images show brain regions with significantly greater signal during interoception compared with autobiographical self conditions. (LH: left hemisphere; RH: right hemisphere).

Exteroception compared with the autobiographical self conditions (traits and facts considered together) showed greater activity bilaterally in a posterior part of the anterior cingulate (i.e., midcingulate) and medial premotor cortex, the most superior part of the PMC (i.e., in the superior precuneus, extending from its anterior limit to its posterior limit), orbitofrontal cortex, basal forebrain, superior and middle frontal gyri adjacent to the precentral sulcus (premotor cortices), the middle and inferior frontal gyrus adjacently to the inferior frontal sulcus; the most inferior part of the precentral gyrus, supramarginal gyrus, superior parietal lobule, and insula (including both anterior and posterior insula) in the left inferior temporal and fusiform gyri, and extrastriate body area (Figure S7, Table S6).


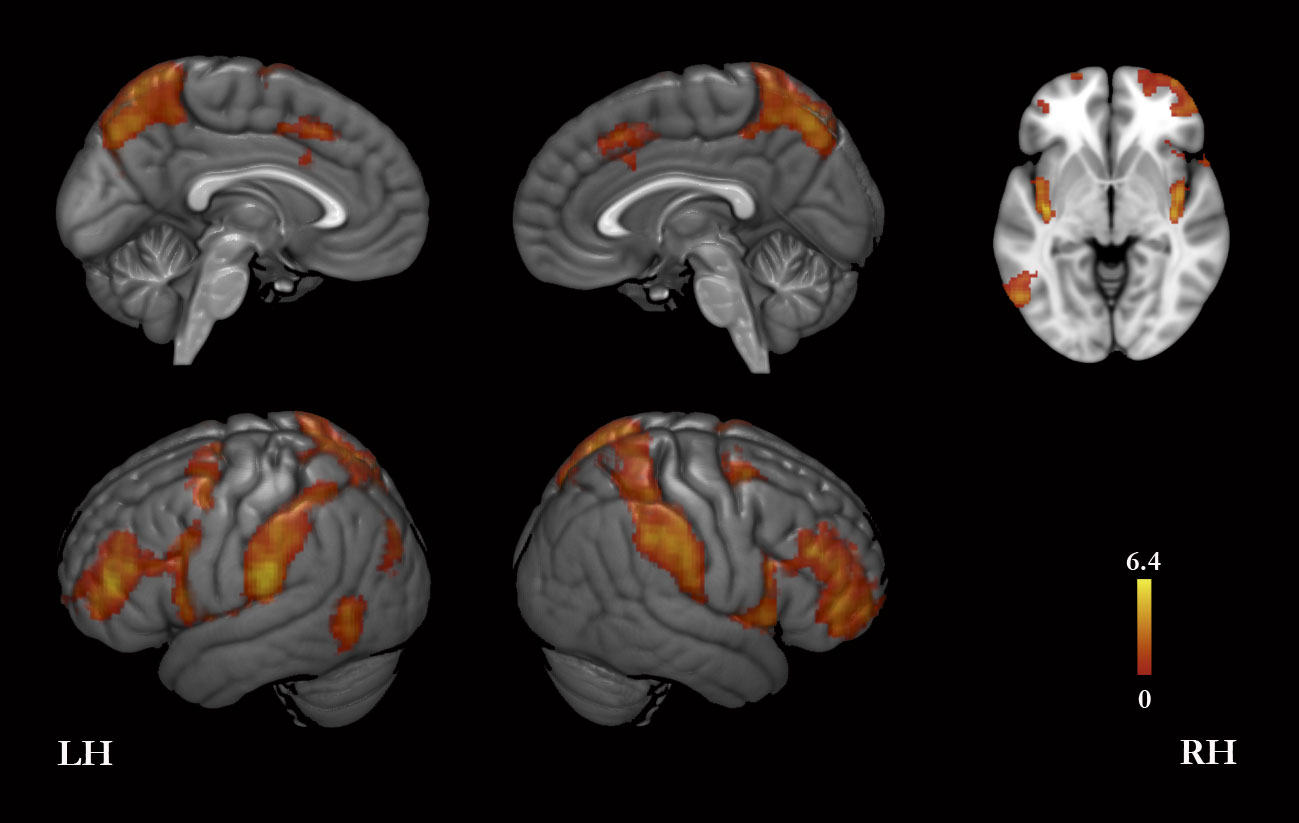


**Figure S7**. Exteroception minus autobiographical self (facts and traits). The images show brain regions with significantly greater signal during exteroception compared with autobiographical self conditions. (LH: left hemisphere; RH: right hemisphere).

**
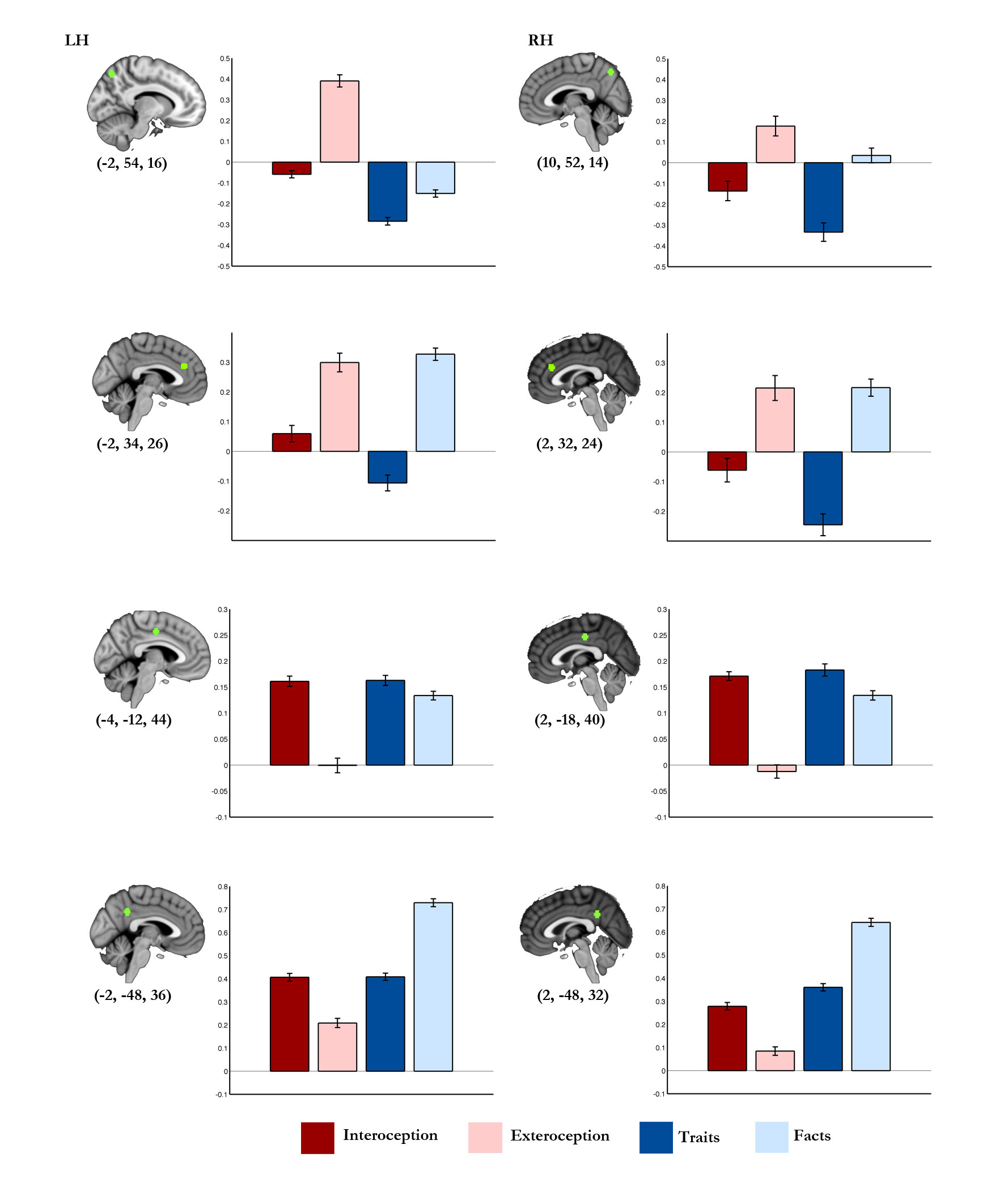
**

**Figure S8**. Parameter estimates for each condition in CMSs. ROIs consisted of spheres of 5mm radius centered on activation peaks for contrasts (Table S6). MNI coordinates (x, y, z) in parentheses; error bars represent SEM.

**
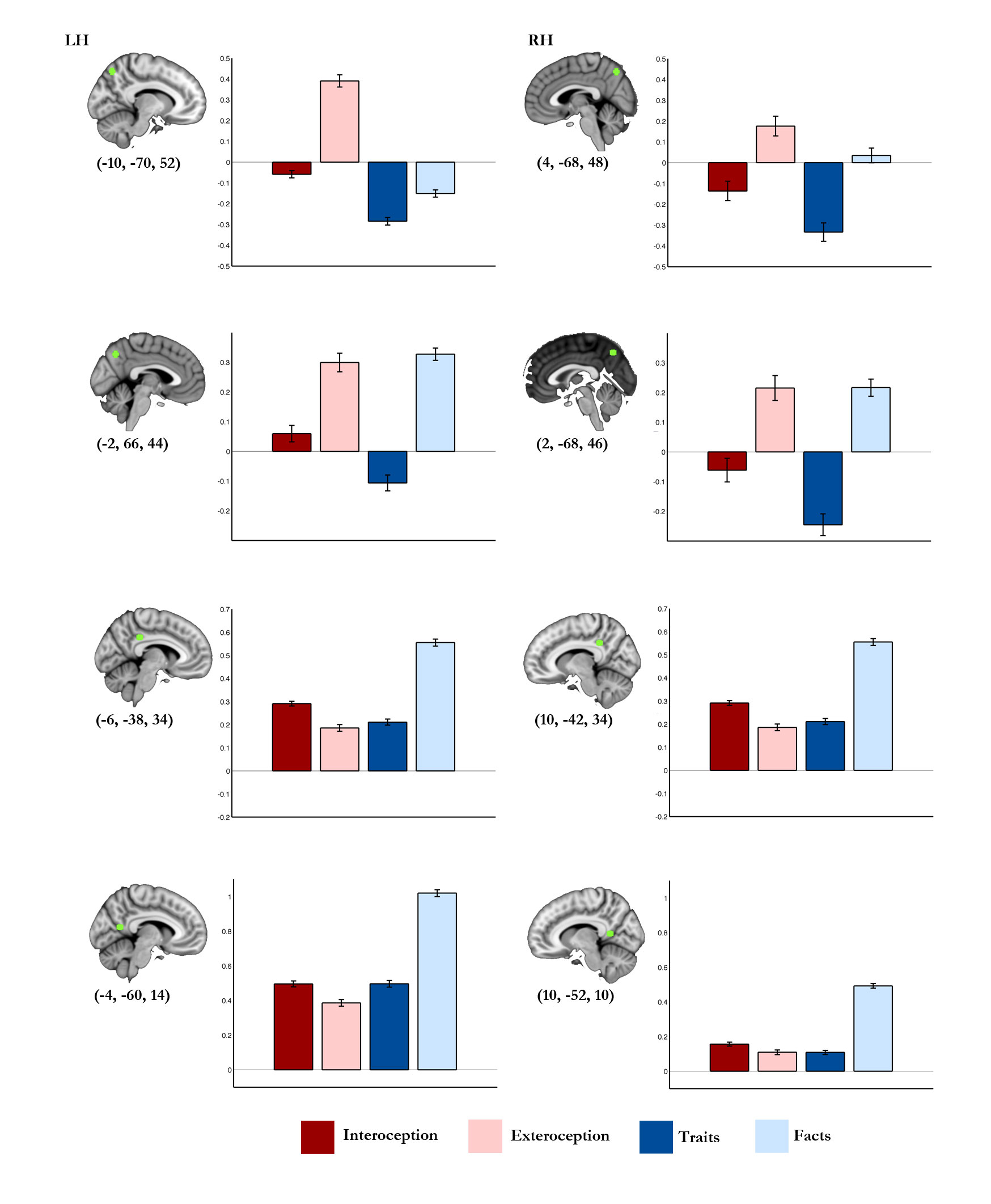
**

**Figure S9**. Parameter estimates for each condition in CMSs. ROIs consisted of spheres of 5mm radius centered on activation peaks for contrasts (Table S6). MNI coordinates (x, y, z) in parentheses; error bars represent SEM.

**Table S1.** Activation peaks for the contrast autobiographical self (facts + traits) minus core-self (interoception + exteroception). Coordinates are in the MNI-152 standard space. (H: hemisphere; L, left; R, right; Z: Z-score).

| **Structure** | **H** | **x** | **y** | **z** | **Z** |
| --- | --- | --- | --- | --- | --- |
| Medial prefrontal cortex/anterior cingulate cortex | L | -6 | 52 | -10 | 5.77 |
|  | R | 2 | 32 | -10 | 6.10 |
| Paracentral gyrus | L | -4 | -26 | 54 | 3.39 |
|  | R | 8 | -34 | 66 | 2.98 |
| Posterior cingulate cortex/ retrosplenial cortex/ precuneus | L | -4 | -54 | 22 | 5.98 |
|  | R | 10 | -52 | 22 | 6.16 |
| Inferior frontal gyrus/ orbitofrontal cortex | L | -42 | 34 | -12 | 4.22 |
|  | R | 40 | 34 | -18 | 4.42 |
| Middle frontal gyrus/ superior frontal gyrus | L | -12 | 26 | 52 | 4.7 |
|  | R | 16 | 44 | 36 | 4.76 |
| Precentral gyrus/ postcentral gyrus | L | -48 | -22 | 62 | 4.16 |
|  | R | 42 | -18 | 64 | 3.63 |
| Temporal pole | L | -54 | 4 | -34 | 5.11 |
|  | R | 40 | 24 | -34 | 4.64 |
| Middle temporal gyrus/ inferior temporal gyrus | L | -60 | -6 | -22 | 5.91 |
|  | R | 60 | -6 | -20 | 5.46 |
| Superior parietal lobule/ angular gyrus | L | -50 | -68 | 36 | 5.46 |
|  | R | 58 | -64 | 38 | 4.58 |
| Middle temporal gyrus/ angular gyrus | R | 60 | -44 | -8 | 4.87 |
| Lateral occipital cortex | L | -24 | -84 | 12 | 4.16 |
|  | R | 40 | 84 | -10 | 4.93 |
| Insula | L | -28 | 12 | -12 | 4.16 |
|  | R | 28 | 18 | -14 | 3.33 |
| Caudate/ putamen/ accumbens | L | 12 | 18 | 10 | 4.4 |
|  | R | -6 | 12 | -6 | 5.09 |
| Thalamus | L/R | 0 | -4 | 10 | 4.39 |
| Hippocampus | L | -24 | -18 | -16 | 4.99 |
|  | R | 22 | -20 | -14 | 5.25 |
| Amygdala | R | 14 | -4 | -16 | 3.35 |

**Table S2.** Activation peaks for the contrast facts minus core-self (interoception + exteroception). Coordinates are in the MNI-152 standard space. (H: hemisphere; L, left; R, right; Z: Z-score).

| **Structure** | **H** | **x** | **y** | **z** | **Z** |
| --- | --- | --- | --- | --- | --- |
| Medial prefrontal cortex/ anterior cingulate cortex | L | -6 | 52 | -10 | 6.57 |
|  | R | 2 | 56 | -8 | 6.31 |
| Paracentral gyrus | L | -2 | -14 | 54 | 3.02 |
|  | R | 4 | -22 | 58 | 2.95 |
| Posterior cingulate cortex/retrosplenial cortex/ precuneus | L | -4 | -58 | 32 | 6.52 |
|  | R | 8 | -52 | 22 | 6.93 |
| Inferior frontal gyrus/ orbitofrontal cortex | L | -44 | 34 | -14 | 4.12 |
|  | R | 40 | 34 | -18 | 4.54 |
| Middle frontal gyrus/ superior frontal gyrus | L | -38 | 18 | 42 | 5.29 |
|  | R | 26 | 26 | 40 | 5.33 |
| Postcentral gyrus | L | -46 | -22 | 62 | 4.07 |
|  | R | 16 | -26 | 54 | 3.84 |
| Precentral gyrus/ postcentral gyrus | L | -30 | -16 | 76 | 3.91 |
|  | R | 16 | -32 | 62 | 3.4 |
| Superior parietal lobule/ lateral occipital cortex/  angular gyrus | L | -48 | -70 | 36 | 6.53 |
|  | R | 42 | -56 | 34 | 5.64 |
| Middle temporal gyrus/ temporal pole | L | -58 | -10 | -20 | 6.7 |
| Middle temporal gyrus/ inferior temporal gyrus | R | 60 | -6 | -16 | 5.96 |
| Temporal pole | R | 40 | 26 | -32 | 5.19 |
| Thalamus | L | -4 | -2 | 6 | 3.94 |
|  | R | 2 | -10 | 6 | 3.72 |
| Caudate/ accumbens | L | -6 | 12 | -6 | 5.01 |
|  | R | 6 | 12 | 6 | 4.31 |
| Hippocampus | L | -24 | -18 | -16 | 5.95 |
|  | R | 22 | -20 | -16 | 5.29 |
| Amygdala | L | -14 | -6 | -16 | 3.61 |
|  | R | 32 | -10 | -16 | 3.93 |
| Insula | L | -28 | -12 | -12 | 3.42 |

**Table S3.** Activation peaks for the contrast traits minus core-self (interoception + exteroception). Coordinates are in the MNI-152 standard space. (H: hemisphere; L, left; R, right; Z: Z-score).

| **Structure** | **H** | **x** | **y** | **z** | **Z** |
| --- | --- | --- | --- | --- | --- |
| Medial prefrontal cortex/ anterior cingulate cortex | L | -4 | 30 | -6 | 4.86 |
|  | R | 8 | 54 | 16 | 5.42 |
| Paracentral gyrus | L | -4 | -26 | 54 | 3.23 |
|  | R | 4 | -32 | 60 | 3.9 |
| Posterior cingulate cortex/ precuneus | L | -4 | -52 | 26 | 4.78 |
|  | R | 6 | -54 | 24 | 4.39 |
| Inferior frontal gyrus/ orbitofrontal cortex | L | -46 | 28 | 2 | 4.07 |
|  | R | 42 | 30 | -20 | 4.04 |
| Precentral gyrus | L | -48 | -24 | 64 | 4.01 |
| Precentral gyrus/ postcentral gyrus | R | 44 | -18 | 66 | 4.16 |
| Postcentral gyrus | L | -42 | -30 | 68 | 4.44 |
| Temporal pole | L | -54 | 4 | -36 | 4.72 |
|  | R | 34 | 28 | -32 | 4.03 |
| Middle temporal gyrus | L | -62 | -12 | -14 | 4.82 |
|  | R | 60 | -6 | -22 | 4.45 |
| Lateral occipital cortex | L | -28 | -88 | 14 | 4.44 |
|  | R | 36 | -90 | -8 | 5.33 |
| Superior parietal lobule/ angular gyrus | L | -54 | -64 | 46 | 3.61 |
| Insula | L | -28 | 12 | 12 | 3.35 |
| Insula/ inferior frontal gyrus | R | 40 | 14 | -14 | 2.42 |
| Hippocampus | R | 22 | -20 | -12 | 3.53 |
| Caudate/ putamen/ accumbens | L | -6 | 14 | -4 | 3.40 |
|  | R | 6 | 10 | -4 | 3.59 |
| Thalamus | L | -2 | -6 | 10 | 3.39 |
|  | R | 2 | -8 | 10 | 3.63 |
| Amygdala | R | 14 | -8 | -12 | 2.88 |

**Table S4.** Activation peaks for the contrast core-self (interoception + exteroception) minus autobiographical self (facts + traits) .Coordinates are in the MNI-152 standard space. (H: hemisphere; L, left; R, right; Z: Z-score).

| **Structure** | **H** | **x** | **y** | **z** | **Z** |
| --- | --- | --- | --- | --- | --- |
| Orbitofrontal cortex | L | -28 | 40 | -18 | 4.98 |
|  | R | 16 | 32 | -16 | 4.34 |
| Precuneus | L | -10 | -76 | 46 | 5.38 |
|  | R | 12 | -74 | 42 | 4.32 |
| Inferior frontal gyrus/ middle frontal gyrus | L | -38 | 40 | 14 | 6.05 |
|  | R | 40 | 42 | 8 | 4.52 |
| Superior frontal gyrus/ precentral gyrus | L | -14 | -4 | 66 | 4.4 |
| Supramarginal gyrus/superior parietal lobule | L | -36 | -48 | 38 | 6.42 |
|  | R | 60 | -40 | 42 | 5.33 |
| Middle temporal gyrus/ lateral occipital gyrus | L | -58 | -64 | 6 | 4.84 |
| Insula | L | -40 | -12 | 4 | 6.05 |
|  | R | 38 | -16 | -4 | 5.12 |

**Table S5**. Activation peaks for the contrast interoception minus autobiographical self (traits and facts). Coordinates are in the MNI-152 standard space. (H: hemisphere; L, left; R, right; Z: Z-score).

| **Structure** | **H** | **x** | **y** | **z** | **Z** |
| --- | --- | --- | --- | --- | --- |
| Precuneus | L | -14 | -72 | 28 | 4.17 |
|  | R | 8 | -30 | 44 | 3.7 |
| Middle frontal gyrus/ inferior frontal gyrus | L | -38 | 38 | 16 | 4.86 |
|  | R | 52 | 50 | 6 | 3.6 |
| Inferior frontal gyrus/ precentral gyrus | L | -54 | -2 | 10 | 5.37 |
|  | R | 60 | 2 | 8 | 4.69 |
| Postcentral gyrus/ supramarginal gyrus | L | -56 | -28 | 32 | 5.21 |
|  | R | 66 | -32 | 44 | 4.87 |
| Middle temporal gyrus/ lateral occipital gyrus | L | -56 | -58 | 4 | 5.23 |
|  | R | 64 | -50 | 10 | 2.39 |
| Superior temporal gyrus | R | 46 | -14 | -8 | 3.25 |
| Inferior temporal gyrus/ temporal pole | R | 38 | -6 | -38 | 3.77 |
| Insula | L | -34 | 8 | 10 | 5.72 |
|  | R | 36 | 10 | 12 | 5.14 |

**Table S6.** Activation peaks for the contrast exteroception minus autobiographical self (traits and facts). Coordinates are in the MNI-152 standard space. (H: hemisphere; L, left; R, right; Z: Z-score).

| **Structure** | **H** | **x** | **y** | **z** | **Z** |
| --- | --- | --- | --- | --- | --- |
| Medial frontal gyrus/ anterior cingulate cortex | L | -2 | 20 | 46 | 4.12 |
|  | R | 4 | 22 | 48 | 4.05 |
| Precuneus/ superior parietal lobule | L | -14 | -72 | 44 | 5.8 |
| Precuneus | R | 12 | -74 | 42 | 5.76 |
| Orbitofrontal cortex | L | -20 | 38 | -18 | 4.92 |
|  | R | 18 | 34 | -18 | 4.23 |
| Basal forebrain | L | -12 | 14 | -14 | 2.97 |
|  | R | 16 | 10 | -18 | 3.02 |
| Superior frontal gyrus/ middle frontal gyrus | L | -24 | 0 | 52 | 4.31 |
|  | R | 30 | 4 | 64 | 4.39 |
| Inferior frontal gyrus/ middle frontal gyrus | L | -38 | 42 | 12 | 6.45 |
|  | R | 42 | 42 | 4 | 5.3 |
| Inferior frontal gyrus/ precentral gyrus | L | -54 | 8 | 6 | 4.64 |
|  | R | 56 | 10 | 4 | 4.5 |
| Inferior temporal gyrus/ fusiform gyrus | L | -44 | -52 | -14 | 4.53 |
| Supramarginal gyrus/superior parietal lobule | L | -62 | -24 | 28 | 6.06 |
|  | R | 58 | -38 | 40 | 5.53 |
| Superior parietal lobule | L | -36 | -48 | 38 | 6.28 |
| Middle temporal gyrus/ lateral occipital gyrus | L | -58 | -64 | 6 | 4.78 |
| Insula | L | -40 | -14 | -4 | 5.55 |
|  | R | 38 | -16 | -4 | 4.95 |

**Table S7.** Activation peaks (and the corresponding contrasts) used for ROI masks of CMSs (H: hemisphere; L, left; R, right; Z: Z-score).

| Contrast | H | x | y | z | Z |
| --- | --- | --- | --- | --- | --- |
| **Medial prefrontal cortex/ anterior cingulate cortex** | | | | | |
|  |  |  |  |  |  |
| Autobiographical self > core self | L | -2 | 54 | 16 | 5.23 |
|  | R | 10 | 52 | 14 | 4.38 |
|  | L | -2 | 58 | -6 | 4.87 |
|  | R | 2 | 58 | -6 | 4.72 |
|  | L | -4 | 36 | -10 | 4.83 |
|  | R | 2 | 32 | -10 | 4.97 |
| Traits > facts | L | -2 | 34 | 26 | 3.91 |
|  | R | 2 | 32 | 24 | 3.97 |
|  |  |  |  |  |  |
| **Posteromedial cortex** | | | | | |
| Core self > autobiographical self | L | -4 | -52 | 58 | 3.40 |
|  | R | 4 | -54 | 58 | 3.13 |
| Autobiographical self > core self | L | -4 | -52 | 26 | 4.78 |
|  | R | 10 | -52 | 22 | 4.46 |
| Exteroception > interoception | L | -10 | -70 | 52 | 4.99 |
|  | R | 4 | -68 | 48 | 5.09 |
| Interoception > exteroception | L | -4 | -12 | 44 | 4.43 |
|  | R | 2 | -18 | 40 | 4.38 |
|  | L | -2 | -48 | 36 | 4.5 |
|  | R | 2 | -48 | 32 | 4.02 |
| Facts > traits | L | -4 | -60 | 14 | 6.34 |
|  | R | 10 | -52 | 10 | 5.63 |
|  | L | -6 | -38 | 34 | 6.24 |
|  | R | 10 | -42 | 34 | 6.08 |
|  | L | -2 | -66 | 44 | 5.78 |
|  | R | 2 | -68 | 46 | 6.03 |
